# Actin-Related Protein 2 (ARP2) and Virus-Induced Filopodia Facilitate Human Respiratory Syncytial Virus Spread

**DOI:** 10.1371/journal.ppat.1006062

**Published:** 2016-12-07

**Authors:** Masfique Mehedi, Thomas McCarty, Scott E. Martin, Cyril Le Nouën, Eugen Buehler, Yu-Chi Chen, Margery Smelkinson, Sundar Ganesan, Elizabeth R. Fischer, Linda G. Brock, Bo Liang, Shirin Munir, Peter L. Collins, Ursula J. Buchholz

**Affiliations:** 1 RNA Viruses Section, Laboratory of Infectious Diseases, National Institute of Allergy and Infectious Diseases, National Institutes of Health, Bethesda, Maryland, United States of America; 2 Division of Pre-Clinical Innovation, National Center for Advancing Translational Sciences, Rockville, Maryland, United States of America; 3 Biological Imaging Section, Research Technologies Branch, National Institute of Allergy and Infectious Diseases, National Institutes of Health, Bethesda, Maryland, United States of America; 4 Microscopy Unit, Rocky Mountain Laboratories, Research Technologies Branch, National Institute of Allergy and Infectious Diseases, National Institutes of Health, Hamilton, Montana, United States of America; Thomas Jefferson University, UNITED STATES

## Abstract

Human respiratory syncytial virus (RSV) is an enveloped RNA virus that is the most important viral cause of acute pediatric lower respiratory tract illness worldwide, and lacks a vaccine or effective antiviral drug. The involvement of host factors in the RSV replicative cycle remains poorly characterized. A genome-wide siRNA screen in human lung epithelial A549 cells identified actin-related protein 2 (ARP2) as a host factor involved in RSV infection. ARP2 knockdown did not reduce RSV entry, and did not markedly reduce gene expression during the first 24 hr of infection, but decreased viral gene expression thereafter, an effect that appeared to be due to inhibition of viral spread to neighboring cells. Consistent with reduced spread, there was a 10-fold reduction in the release of infectious progeny virions in ARP2-depleted cells at 72 hr post-infection. In addition, we found that RSV infection induced filopodia formation and increased cell motility in A549 cells and that this phenotype was ARP2 dependent. Filopodia appeared to shuttle RSV to nearby uninfected cells, facilitating virus spread. Expression of the RSV F protein alone from a plasmid or heterologous viral vector in A549 cells induced filopodia, indicating a new role for the RSV F protein, driving filopodia induction and virus spread. Thus, this study identified roles for ARP2 and filopodia in RSV-induced cell motility, RSV production, and RSV cell-to-cell spread.

## Author Summary

RSV is the most common viral cause of severe acute pediatric lower respiratory tract illness, including pneumonia and bronchiolitis, in young children worldwide. In a genome-wide siRNA screen in human lung epithelial A549 cells infected with RSV expressing green fluorescent protein (RSV-GFP), we identified ARP2 as a cellular factor with a role in the RSV replicative cycle. ARP2 is part of the actin-related protein 2/3 (ARP2/3) complex, which contributes to cell shape and motility through its role in actin polymerization. ARP2 depletion reduced the production and spread of RSV in human lung epithelial cell cultures, with the most noticeable effects at late time points after RSV infection. RSV infection induced the formation of slender actin-rich cell protrusions, called filopodia and increased cell motility. Filopodia formation and cell motility were inhibited by ARP2 knockdown. The filopodia appeared to shuttle RSV to neighboring cells, facilitating virus spread. Thus, RSV uses two previously unrecognized ARP2 dependent features to facilitate viral spread, namely cell motility and filopodia formation.

## Introduction

RSV is the most important viral cause of severe acute pediatric lower respiratory tract illness worldwide, and also causes substantial morbidity and mortality in the elderly as well as in individuals with severe immunosuppression or cardiopulmonary disease. Despite its recognized importance, and despite decades of research, there is no licensed vaccine or specific antiviral therapy.

RSV is an enveloped virus of the family *Pneumoviridae* [1], and contains a single-stranded non-segmented negative-sense RNA genome (approximately 15.2 kb) with 10 genes encoding 11 proteins, including the nucleoprotein N, phosphoprotein P, matrix protein M, RNA dependent RNA polymerase L, transcription factor and second matrix protein M2-1, polymerase factor M2-2 that is expressed from a second open reading frame (ORF) in the M2 mRNA, fusion glycoprotein F, attachment glycoprotein G, small hydrophobic surface protein SH, and nonstructural accessory proteins NS1 and NS2 [2]. RSV infection starts with cellular receptor binding mediated by G and F [3]. The chemokine receptor CX3CR1 has recently been identified as a receptor molecule for the RSV G protein on respiratory epithelial cells [4]. Entry of RSV is not completely defined and may involve cell surface fusion as well as macropinocytosis followed by fusion [5], mediated by the F protein. RSV transcription and replication occur in the cytoplasm, probably in large, dense cytoplasmic inclusion bodies. Progeny virions bud from the plasma membrane [2,6]. In the natural human host, RSV infects respiratory epithelial cells [7].

We recently performed a genome-wide siRNA screen of more than 20,000 genes in human airway epithelial A549 cells infected with RSV-GFP to identify genes that affected viral expression of GFP and therefore may affect the RSV replicative cycle. This survey, which is still in progress and will be published separately, provided presumptive evidence that knockdown of the *ACTR2* gene, which encodes ARP2, resulted in a reduction of viral GFP expression, suggesting that the ARP2 protein promotes RSV infection (For simplicity, we will refer to the *ACTR2* gene and mRNA by the same name as used for the protein, ARP2).

ARP2 is part of the ARP2/3 complex, which plays a central role in actin polymerization [8]. Actin is a major component of the cytoskeleton, and actin rearrangement affects a multitude of intra- and intercellular processes including cell shape, structure, and motility [9]. Actin is present in globular monomeric (G-actin) and polymeric filamentous (F-actin) forms. F-actin can form polymeric structures resulting in cell membrane extensions, such as lamellipodia (sheet-like extensions), filopodia and microvilli (finger-like protrusions), and dot-like podosomes [10]. Cellular actins also appear to be involved in RSV gene expression, replication and morphogenesis [11–13], but the mechanim is poorly understood, and the role of ARPs has not been studied. In the present study, we show that ARP2 knockdown reduced viral gene expression and protein production, viral yield, and cell-to-cell spread in A549 cells. These effects were most prominent at later times of infection, affecting viral spread rather than early events in RSV infection. We found that the RSV infection induces the formation of filopodia on the cell surface, which are distinct from structures representing filamentous RSV particles. Filopodia appear to shuttle RSV particles to neighboring cells, a previously unknown mechanism for RSV spread. We also found that RSV infection increases cell motility, likely contributing to cell-to-cell spread. Our results show that ARP2 contributes to RSV cell-to-cell spread in human lung epithelial cells.

## Results

### The effects of ARP2 knockdown were not evident early in infection

**Fig 1:**
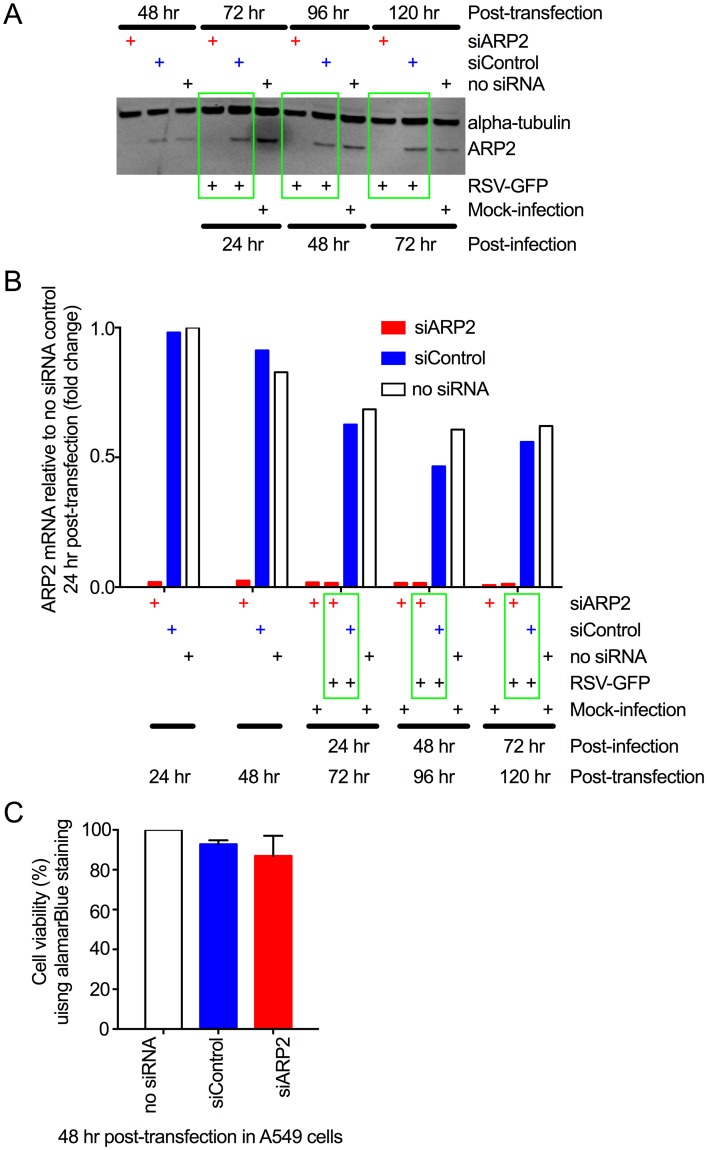
ARP2 knockdown in human respiratory epithelial A549 cells. **(A) Knockdown of ARP2 protein expression.** Replicate monolayers of A549 cells were transfected with siARP2, siControl, or no siRNA, as indicated. At 48 hr post-transfection, one set of monolayers was harvested. The others were infected with RSV-GFP (MOI = 1) or mock-infected, and harvested at 24, 48, and 72 hpi, as indicated. The cells were processed for Western blotting. ARP2 protein was detected using a primary rabbit mAb and an anti-rabbit IgG IRDye800 secondary Ab. Alpha-tubulin, as a loading control, was detected with a primary mouse mAb and an anti-mouse IgG IRDye680 secondary Ab. Bound antibodies were visualized by infrared fluorescence. One representative of four independent experiments is shown. **(B) Knockdown of ARP2 mRNA expression.** A549 cells were transfected with siARP2, siControl, or no siRNA, as indicated. Sets of monolayers were harvested 24 and 48 hr post-transfection. The remaining monolayers were infected with RSV-GFP (MOI = 1) or mock-infected, and harvested at 24, 48, and 72 hpi, as indicated. Total cell-associated ARP2 mRNA was quantified by real-time PCR using a TaqMan assay for ARP2. 18S ribosomal RNA was used as an endogenous control for normalization of each reaction, and the values of ARP2 expression are shown as fold-change relative to the no-siRNA control 24 hr post-transfection. Each sample was tested in quadruplicate by TaqMan assay and the averages are presented. **(C) ARP2 knockdown did not reduce cell viability.** A549 cells were transfected with siARP2, siControl or no siRNA for 48 hr. Cell viability was compared using alamarBlue and expressed relative to the no-siRNA control. Data from three independent experiments, each done in triplicate were combined for analysis. Error bars show standard deviation (SD).

**Fig 2:**
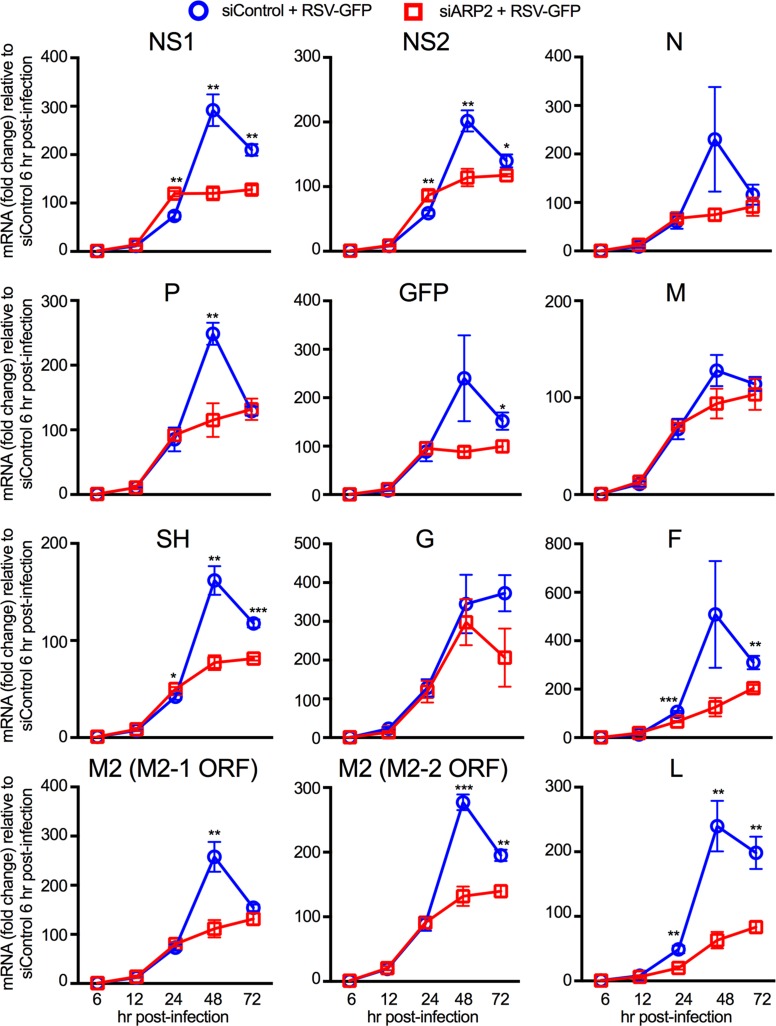
ARP2 knockdown reduced RSV mRNAs late after infection. A549 cells were transfected with siARP2 (open red squares) or siControl (open blue circles) for 48 hr followed by infection with RSV-GFP (MOI = 1) for 72 hr. Total mRNA for the eleven RSV ORFs and GFP was quantified at 6, 12, 24, 48 and 72 hpi by qRT- PCR using customized ORF-specific TaqMan assays. Beta-actin was used as an endogenous control against which each mRNA value was normalized, and the fold change of each RSV mRNA was quantified relative to the siControl-transfected samples at 6 hpi. One representative of two independent experiments is shown. Each experiment was performed in three biological replicates and qRT-PCR for each sample was done in quadruplet. Statistical significance analysis was done using a one-tailed unpaired t-test with the assumption of equal variance. *, p<0.05; **, p<0.01, ***, p<0.001. Error bars: standard error of mean.

**Fig 3:**
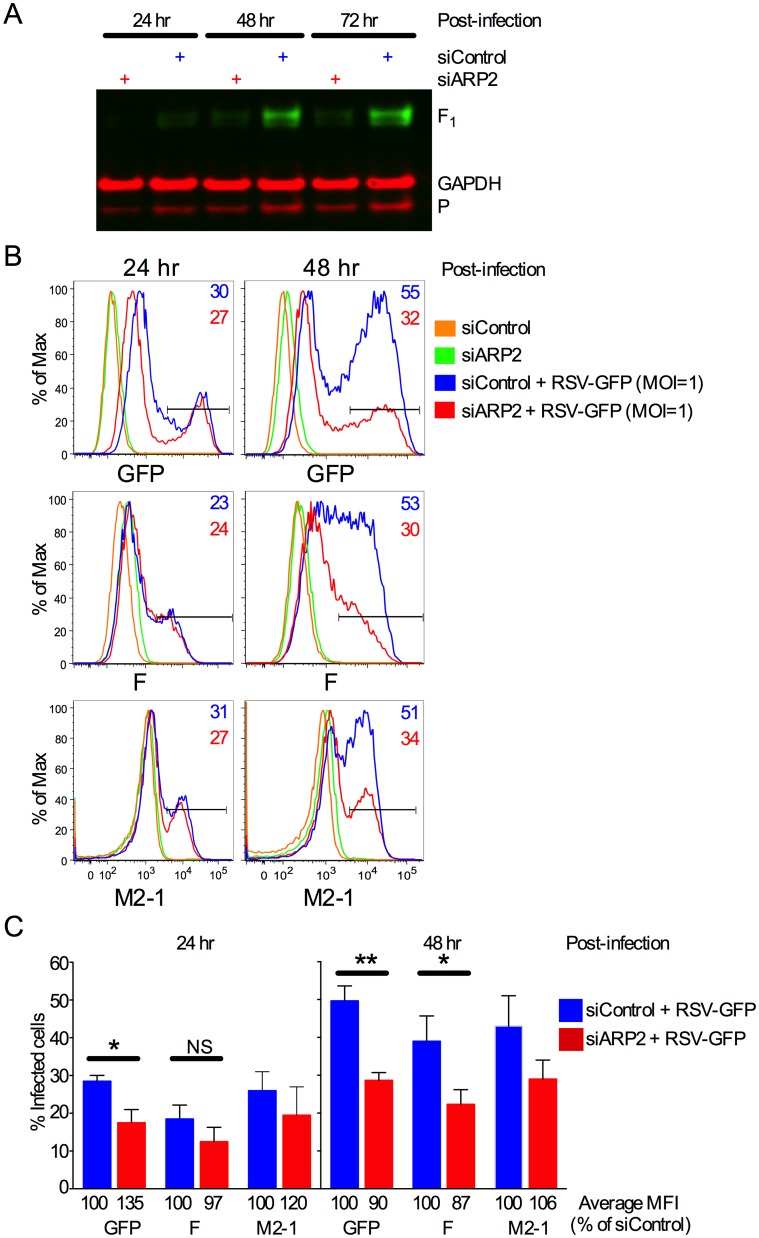
ARP2 knockdown reduced RSV protein expression and the number of infected cells late after RSV infection. **(A) Western blot analysis.** RSV F1 protein (48 kDa) and P protein (33 kDa) were detected by Western blot analysis performed on cell lysates from the experiment described in Fig 2. RSV F was detected using a primary mouse mAb and an IRDye 800CW conjugated goat anti-mouse secondary Ab; GAPDH as a loading control was detected using a rabbit pAb and an IRDye 680RD conjugated goat anti-rabbit IgG (H+L) Ab. The same membrane was reprobed for RSV P using a primary mouse mAb and an IRDye 680RD conjugated goat anti-mouse Ab. **(B, C) Percentage and mean fluorescence intensity (MFI) of infected cells measured by multicolor flow cytometry.** A549 cells were transfected with siARP2 or siControl for 48 hr followed by infection with RSV-GFP (MOI = 1) or mock-infection. Cells were harvested 24 or 48 hpi, stained with Live/Dead dye, fixed, permeabilized and immunostained with mAbs specific to the F and M2-1 proteins as described in Materials and Methods. Single live cells that were positive for GFP, F, and M2-1 were quantified at 24 and 48 hpi, with the gating shown by the horizontal bars. (B) Histograms for GFP-, F-, and M2-1-expressing cells at 24 or 48 hpi. Gates for GFP-, F-, and M2-1-positive cells are shown, and the percentage of positive cells is indicated in the top right corner of each individual histogram. (C) Mean percentage of GFP-, F-, or M2-1-positive cells at 24 and 48 hpi from several experiments. The corresponding mean fluorescence intensities (MFI) of the gated populations are indicated below each bar. Data for GFP- and F-expressing cells are from three independent experiments and data of M2-1-expressing cells are derived from two independent experiments. Error bars: standard error of mean. *, p<0.05; **, p<0.01, NS, not significant; two-tailed t-test.

To investigate the effects of ARP2 knockdown on RSV infection, A549 cells were transfected with an ARP2-specific siRNA (siARP2) or a negative control siRNA (siControl). At 48 hr post-transfection, the cells were infected with RSV-GFP (that expresses GFP from an additional gene [14]) at a multiplicity of infection (MOI) of 1.0 plaque forming unit (PFU)/cell. The cultures were evaluated for ARP2 and viral gene expression at 0, 24, 48, and 72 hr post-infection (hpi) (Figs 1–3). To put this timing into context, in a growth cycle for RSV in A549 cells (which is shown later), the production of progeny virus was first detected at approximately 12 hpi, increased substantially by 24–48 hpi, and was maintained or slowly increased through approximately 72 hpi.

The effectiveness of siARP2-mediated knockdown on ARP2 protein expression was evaluated by Western blot analysis (Fig 1A). A substantial reduction in ARP2 protein accumulation was observed 48 hr following siARP2 transfection, the time of RSV infection, and remained stable for the duration of the 72 hr infection. Quantitative (q) RT-PCR confirmed that knockdown of ARP2 mRNA was highly efficient and stable over the same time course (Fig 1B). In the absence of siARP2, there was no significant difference in the expression of ARP2 mRNA or protein in RSV-GFP infected versus uninfected cells, indicating that expression was unaffected by RSV infection (Fig 1A and 1B). Importantly, ARP2 knockdown was achieved without compromising cell viability, as determined by alamarBlue viability assay (Fig 1C).

As a first step to investigate the effects of ARP2 knockdown on RSV infection, we used qRT-PCR to quantify the accumulation of the complete set of mRNAs encoded by RSV-GFP in A549 cells transfected with siARP2 or siControl (Fig 2). The input MOI in this experiment was 1 PFU/cell (calculated by titration on Vero cells). Since the susceptibility of A549 cells is slightly lower than that of Vero cells, the majority (about 2/3) of A549 cells was not infected by the initial inoculum and could support subsequent rounds of replication. Each of the RSV ORFs was quantified, including M2-1 and M2-2 that are expressed on a single mRNA (Fig 2). This showed that, for the first 24 hr following infection, the accumulation of each virally-encoded mRNA was similar in ARP2-knockdown and control cells, except for the RSV F and L mRNA levels which were slightly but significantly reduced in siARP2 depleted cells; unexpectedly, the NS1 and NS2 mRNA levels were slightly but significantly lower in siControl treated cells than in siARP2 cells. However, between 24 and 48 hpi, there was a much smaller increase in the accumulation of viral mRNAs in the ARP2-knockdown cells than in the control cells. By 48 hpi, NS1, NS2, P, SH, M2 and L mRNA levels were significantly lower in siARP2-knockdown cells; the difference in P mRNA levels as well as M2 mRNA levels measured using the M2-1 ORF specific assay were significant only at 48 hr, and the differences in GFP and F mRNA levels reached significance by 72 hpi. N, M, G mRNA levels also were lower in siARP2 transfected A549 cells at late time points after infection, but the differences to siControl transfected cells were not statistically significant.

Western blot analysis showed that the accumulation of viral proteins was also reduced, shown here for P and F (Fig 3A). For a more sensitive analysis of protein expression over time, the kinetics of infection and viral protein expression in siARP2-transfected cells were further investigated by flow cytometry using expression of GFP, F, and M2-1 as markers for infection. Data from a representative experiment are shown in Fig 3B. At 24 hpi, we found little difference in the percentage of RSV-infected A549 cells in ARP2-knockdown versus control cultures. In contrast, at 48 hpi, the number of GFP-, M2-1-, or F-expressing cells had substantially increased in the control cultures, while only a small increase was observed in the siARP2-treated cultures. A similar pattern for the percentage of infected cells was observed when the results were averaged from two (M2-1) or three (GFP and F) independent experiments (Fig 3C). In addition, Fig 3C shows that the average mean fluorescence intensity (MFI) for each of these proteins was similar between siControl- and siARP2-transfected cells at both 24 and 48 hpi. These results show that ARP2 knockdown did not reduce RSV protein expression in individual infected cells; rather, ARP2 knockdown reduced the spread of RSV infection at late time points after infection.

**Fig 4:**
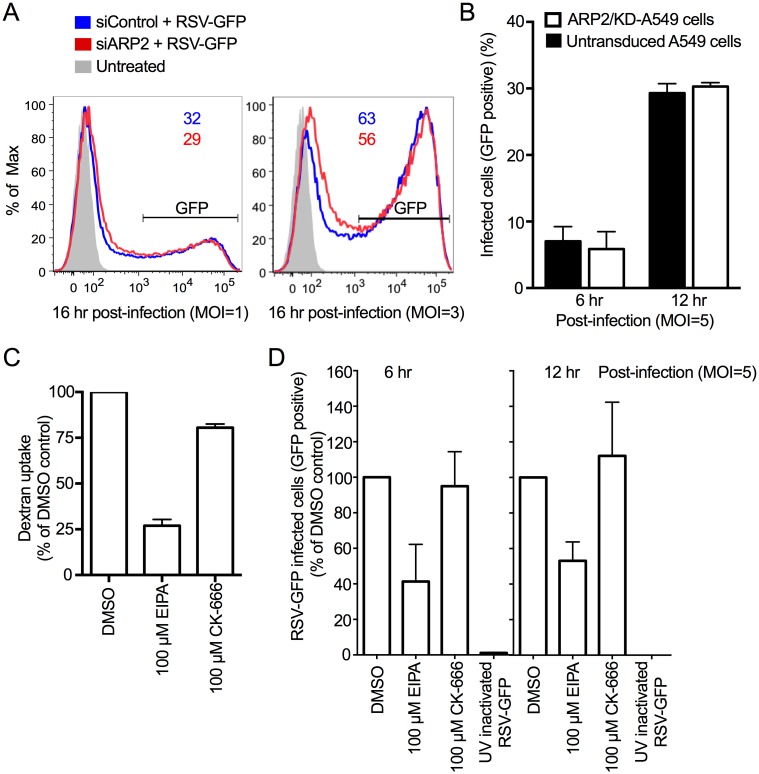
ARP2 knockdown did not reduce RSV entry. **(A) GFP expression by RSV-GFP at 16 hpi was unaffected by ARP2 knockdown.** A549 cells were transfected with siARP2 or siControl for 48 hr followed by infection with RSV-GFP (MOI = 1, left, or MOI = 3, right). At 16 hpi, cells were harvested, stained with Live/Dead dye and, fixed, and the number of single live GFP-expressing cells was quantified by flow cytometry. Untreated cells (no transfection, no infection) were processed in parallel and are shown in grey. The percentage of positive cells is indicated at the top of each histogram **(B) GFP expression by RSV-GFP was unaffected at 6 and 12 hpi in an A549 cell line with constitutive ARP2-knockdown.** ARP2/KD-A549 cells (i.e., A549 cells with constitutive ARP2 knockdown) and untransduced cells were infected with RSV-GFP (MOI = 5) for 6 and 12 hr. Cells were harvested, stained with Live/dead dye, then fixed, and GFP expression was used to quantify the percentage of live single infected cells by flow cytometry. **(C) The macropinocytosis inhibitor EIPA reduced dextran uptake in A549 cells.** A549 cells were treated with 100 μM EIPA, 100 μM CK-666, or DMSO (solvent control) for 1 hr, followed by incubation with dextran fluorescein (1mg/ml, as a marker for uptake by macropinocytosis) for 30 min. Cells were harvested, stained with Live/Dead dye, and fixed. The MFI of single live dextran fluorescein-treated cells was quantified by flow cytometry and expressed as the percent of DMSO control sample used as a reference. **(D) The macropinocytosis inhibitor EIPA, but not the ARP2/3 complex-driven actin nucleation inhibitor CK-666, blocks RSV entry.** A549 cells were treated with 100 μM EIPA, 100 μM CK-666, or DMSO (solvent control) for 1 hr followed by infection with RSV-GFP (MOI = 5) for 6 hr or 12 hr. Cells were harvested, stained with Live/Dead dye, and fixed. The number of single live infected cells was quantified by flow cytometry based on GFP expression. At least 100,000 single live cells were acquired per sample. B, C and D show combined data from two independent experiments. Error bar: SD.

To exclude a possible role of ARP2 in RSV entry, A549 cells were studied at earlier time points after infection (Fig 4). Flow cytometry showed that, at 16 hpi, the proportion of GFP-expressing cells in cultures infected at an MOI of 1 or 3 was similar in siARP2- and siControl-treated cells (Fig 4A). To exclude possible artifacts due to transfection, a stable ARP2-knockdown A549 cell line (ARP2/KD-A549 cells) was generated using a lentiviral vector system expressing three small hairpin RNAs to ARP2, and RSV-GFP infection was analyzed at 6 or 12 hpi by flow cytometry using GFP expression as a marker for infection. We found that the proportion of infected (ARP2/KD-A549 cells) did not differ from that in untransduced A549 cells, even at higher MOIs (e.g. MOI = 5) (Fig 4B). These results suggested that ARP2 does not have a role at early stages in RSV infection.

Next, we evaluated a possible role for ARP2 in RSV entry using the compound CK-666, which is an inhibitor of ARP2/3 complex-driven actin nucleation that acts by stabilizing the ARP2/3 complex in an inactive conformation [15] (Fig 4C and 4D). For comparison, we also evaluated EIPA (5-ethylisopropyl amiloride), which is a potent inhibitor of macropinosome formation and has been demonstrated to inhibit RSV entry [5], and thus is a positive control for the inhibition of entry. A preliminary dose-ranging experiment was performed in which A549 cells were incubated for 1 hr with various concentrations of EIPA, followed by 30 min further incubation with 1 mg/ml dextran fluorescein, which is a marker for uptake by macropinosomes [16]). Dextran uptake was quantified by flow cytometry. This identified 100 μM EIPA as the optimal concentration, reducing dextran uptake by 75%, whereas 100 μM of CK-666 (the known effective concentration [17,18]) or DMSO solvent had no substantial effect on dextran uptake (Fig 4C). Live/Dead cell staining indicated a lack of cytotoxicity on A549 cells at this concentration. Therefore, A549 cells were incubated with 100 μM EIPA, 100 μM CK-666, or DMSO (solvent-only) for 1 hr, followed by RSV-GFP infection (MOI = 5) for 6 or 12 hr, followed by analysis of GFP expression by flow cytometry (Fig 4D). The EIPA treatment reduced the number of infected cells by about 60% at 6 hpi or 50% at 12 hpi, whereas CK-666 and the DMSO control had little effect (Fig 4D). These results confirm that ARP2 does not detectably affect RSV entry or gene expression in A549 cells during the first 6–16 hpi.

### ARP2 knockdown reduced the production of progeny RSV particles, but did not reduce the ability of RSV to form syncytia

**Fig 5:**
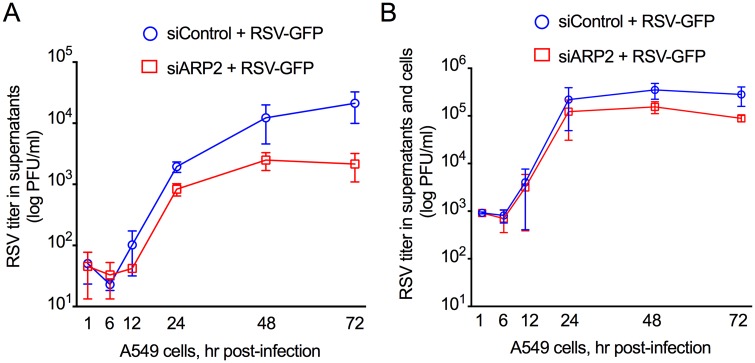
ARP2 knockdown reduced production of infectious RSV. From the time course experiment described in Figs 1A, 2 and 3A, replicate wells of A549 cells were transfected with siARP2 or siControl, incubated for 48 hr, and infected with RSV-GFP (MOI = 1), and samples were harvested at the indicated time, and viral titers were quantified by plaque assay visualized by immunostaining [61]. **(A)** Virus titers were measured in clarified tissue culture medium harvested from infected cell culture without disturbing the cell monolayer (released virus); and **(B)** virus titers were measured in clarified tissue culture medium from infected cell cultures in which the cells had been scraped into the medium and vortexed to release cell-associated virus (cell-associated virus plus released virus). A and B show combined data from two independent experiments, each performed in triplicate. Error bar: standard error of mean.

We also investigated the production of infectious virus in response to ARP2 knockdown by siARP2, which was done as part of the experiments described in Figs 1A, 2 and 3A. In one set of cultures, we harvested the cell culture medium alone without disturbing the cells to measure released virus over a 72 hr period. This showed that ARP2 knockdown resulted in a ~10-fold reduction in the production of free infectious virus in the supernatant (Fig 5A). In a second set of cultures, the infected cells were scraped into the medium and the suspension was vortexed and clarified in order to quantify total virus production (i.e., cell-associated and cell-free released virus, Fig 5B). The titer of infectious progeny RSV was reduced in the ARP2-knockdown cultures compared to the control cells, but this difference was smaller for total RSV (Fig 5B) than for released RSV (Fig 5A). We then evaluated the effect of ARP2 knockdown on RSV production in another human airway epithelial cell line, Calu-3 cells [19]. Similar to the results in A549 cells, we found that ARP2 knockdown did not reduce cell viability, and knockdown was stable up to 120 hr post siRNA-transfection (S1 Fig). In the ARP2 depleted Calu-3 cells, production of free infectious virus was reduced at late time points after infection, and the production of total infectious progeny was reduced by about 10 fold at 72 hpi, a greater reduction than that observed in A549 cells (S1 Fig).

In an additional experiment, we included human parainfluenza virus type 3 (HPIV3) expressing GFP (HPIV3-GFP) for comparison (S2A Fig). Replicate cultures of A549 cells were transfected with siARP2 or siControl, infected with RSV-GFP or HPIV3-GFP, and harvested at 24, 48, and 72 hpi. Quantification of free infectious progeny virus in the supernatant showed that the production of released RSV-GFP was reduced by ~10-fold in siARP2-transfected cells compared to siControl-transfected cells, consistent with the results in Fig 5A. In contrast, there was little or no reduction in the release of HPIV3-GFP in siARP2 treated cells, indicating that the effect was specific to RSV.

To evaluate the effects of ARP2 knockdown on RSV-induced syncytium formation, the RSV-GFP-infected cell monolayers from the experiment described immediately above (harvested at 24, 48, and 72 hpi) were fixed, permeabilized, and stained with the nuclear fluorescent stain diamidino-2-phenylindole (DAPI) and with rhodamine phalloidin to visualize the actin cytoskeleton. The total coverslip was scanned by fluorescence microscopy, and at least 5000 cells per treatment were analyzed in each of two independent experiments to quantify the number of nuclei that were present in GFP-positive syncytia (syncytia were defined as containing ≥ 3 nuclei) compared to the total number of nuclei in all RSV-infected cells (whether in syncytia or not). We detected a significant reduction in the overall number of RSV-GFP infected cells in siARP2-knockdown cells (consistent with the results in Fig 3B), but, when corrected for the lower rate of infected cells present in the evaluated fields, syncytium formation was not reduced in siARP2 treated cells (S2B Fig). Thus, ARP2 does not seem to have a role in RSV fusion and syncytia formation.

### Effects of ARP2 knockdown on RSV budding and morphology

**Fig 6:**
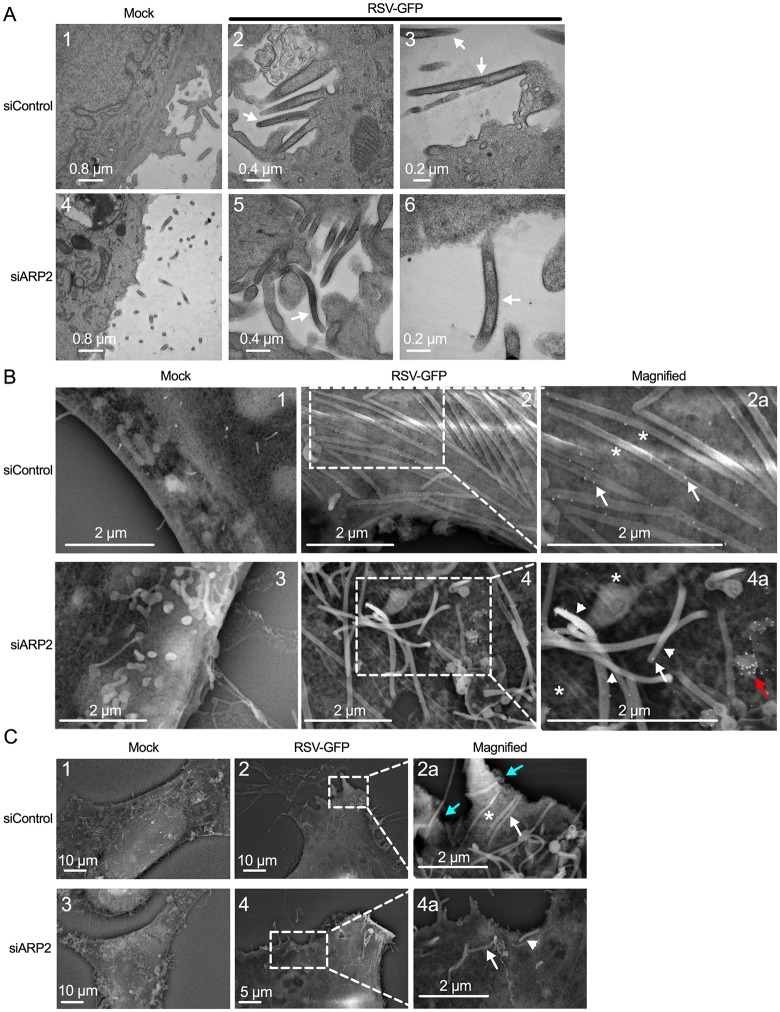
ARP2 knockdown reduced RSV budding with no apparent change in virus morphology. **(A) TEM.** A549 cells were transfected with siARP2 (bottom panels) or siControl (top panels) for 48 hr followed by mock infection (left panels) or infection with RSV-GFP (MOI = 1, panels 2, 3, 5, 6) for 24 hr (the expression of GFP is incidental to this particular experiment). Cells were fixed with glutaraldehyde. The cell surface was evaluated. Virus filaments are indicated with arrows. Nominal magnifications are 6000x, 12000x, 15000x, 6000x, 12000x, and 25000x for panels 1, 2, 3, 4, 5 and 6, respectively. The arrows indicate examples of presumptive RSV virions. **(B) Immuno-SEM.** A549 cells were transfected with siARP2 (bottom panels) or siControl (top panels) for 48 hr followed by mock infection (panels 1, 3) or infection with RSV-GFP (MOI = 5, panels 2 and 4; areas marked are enlarged in 2a and 4a) for 24 hr (the expression of GFP is incidental to this particular experiment). Cells were fixed with glutaraldehyde. RSV F protein was labeled with an anti RSV F mAb, followed by goat anti-mouse secondary Ab conjugated to 15 nm gold beads. Examples of cell-associated virus filaments are indicated with asterisks, and examples of particles that appeared to be lifted off the surface in a disordered array are indicated with arrowheads. Examples of F-specific immunogold particles on filaments are indicated with arrows, and an example of a small surface protuberance that stained heavily with F-specific immunogold is indicated with a red arrow. **(C)** Lower-magnification images from the same experiment as part B and cellular extensions (presumptively lamellipodia or filopodia) are shown in *cyan* arrows. The white arrow and arrowhead are as in part B.

We investigated the effects of ARP2 knockdown on RSV particle morphology using transmission electron microscopy (TEM) (Fig 6A). ARP2-transfected- or siControl-transfected A549 cells were mock-infected or infected with RSV at an MOI of 1, and were fixed at 24 hpi. Overall, areas with accumulations of particles were substantially less frequent on ARP2-knockdown infected cells, and therefore we scanned for areas with accumulations of budding particles and made photomicrographs of these areas to compare virion morphology. In Fig 6A, panels 2 and 3 (siControl-knockdown infected cells) and 5 and 6 (siARP2-knockdown infected cells) show representative examples of cell-associated virions (indicated by arrows). This comparison indicated that, while the abundance of particles was reduced in infected cells treated with siARP2, in those areas where virions were evident there were no apparent changes in virion morphology between infected cells treated with siARP2 versus siControl.

Next, we examined cell surface morphology and virion budding by immune scanning electron microscopy (immuno-SEM) (Fig 6B and 6C). A549 cells were transfected with siARP2 or siControl, and infected with RSV at an MOI of 5 or mock-infected. At 24 hpi, the cells were fixed and subjected to immunostaining with a primary mouse monoclonal antibody (mAb) against the F protein and a secondary anti-mouse-IgG polyclonal antibody (pAb) conjugated with gold particles (15 nm), and analyzed by SEM. As shown in Fig 6B, panels 2 and 2a, the surface of the siControl-transfected RSV-infected cells contained areas with large numbers of long filamentous structures (asterisks) that were organized in parallel arrays that appeared to be lying flat on the infected cell surface. These structures were not evident on the surface of siControl-transfected mock-infected cells (Fig 6B, panel 1). Furthermore, these structures stained with F-specific immunogold (Fig 6B, panel 2a, arrows), whereas images of siControl-transfected mock-infected cells examined in parallel exhibited a lack of staining. The dimensions of these filamentous structures were approximately 100 nm in diameter and 2–10 μm in length, which is consistent with values reported for RSV virions [20]. Thus, these appeared to be filamentous progeny virus particles.

The surface of the siARP2-transfected RSV-infected cells showed a reduced number of presumptive viral filaments, suggesting that ARP2 knockdown reduced RSV budding (Fig 6B, panels 4 and 4a). Some of these particles appeared to lie on the cell surface (Fig 6B, panels 4 and 4a, asterisks), comparable to what was observed in siControl-transfected RSV-infected cells, whereas a number of particles appeared to be lifted off the surface in a disordered array (indicated by arrowheads). In addition, the surface of siARP2-transfected infected cells contained occasional small surface protuberances that stained heavily with F-specific immunogold and might represent aberrant, incomplete budding of particles containing F protein (Fig 6B, panel 4a, red arrow). Thus, while TEM suggested that ARP2 knockdown did not visibly alter the morphology of the RSV particle, immuno-SEM suggested that ARP2 knockdown resulted in virus filaments that were somewhat disorganized at the cell surface, as well as in smaller protuberances that stained well for F protein.

Lower-magnification images of this same experiment revealed another difference between mock-infected and RSV-infected cells. Specifically, siControl-transfected RSV-infected cells were observed to contain prominent surface protrusions (Figs 6C and S3, panel 2, cyan arrows) that were largely absent and reduced in length in siControl-transfected mock-infected cells (Figs 6C and S3, panel 1). These protrusions also were largely absent in siARP2-transfected RSV-infected cells (Figs 6C and S3, panel 4). The size and nature of these protrusions identified them presumptively as lamellipodia and filopodia, which are cytoplasmic extensions involved in cell motility, sensing, and cell-to-cell interactions [21]. Lamellipodia typically are broad flat cytoplasmic protrusions containing an internal branched actin network, and filopodia are slender cytoplasmic protrusions that extend beyond the leading edge of lamellipodia and contain linear actin filaments (F-actin), reviewed in [22]. The filopodia are investigated further below.

### RSV infection induced filopodia, which also involved ARP2

**Fig 7:**
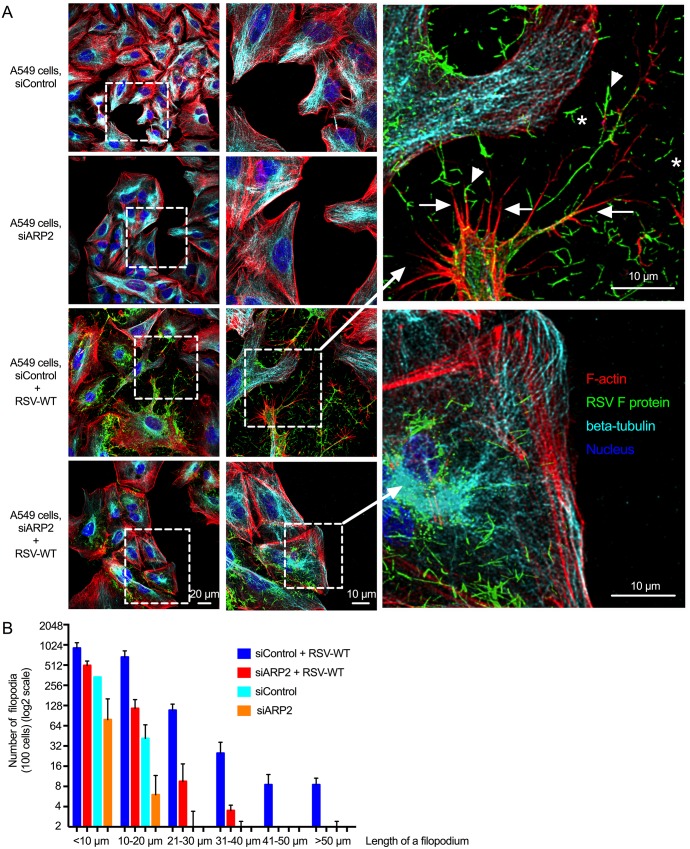
RSV infection induced the formation of filopodia, and ARP2 knockdown reduced the number of long RSV-induced filopodia. **(A) RSV-induced filopodia.** A549 cells were transfected with siARP2 or siControl for 48 hr followed by mock infection or infection with RSV-WT (MOI = 1) for 24 hr. Cells were fixed, permeabilized, and immunostained for RSV F (*green*) and beta-tubulin (*cyan*, here a pseudocolor) by incubating with mouse mAb for F protein and rabbit mAb for beta-tubulin followed by the secondary antibodies ant-mouse AlexaFluor_488_ and anti-rabbit AlexaFluor_647_ respectively. The cells were further stained with rhodamine phalloidin (*red*) to detect F-actin and with the nuclear stain DAPI (*blue*). The images show all four colors (versions that individually show *green*, *cyan*, and *red* are shown in S4 Fig). In the enlargement, examples of filopodia (*red*) are indicated with arrows, examples of what appear to be released filamentous RSV particles (*green*) are indicated with asterisks, and examples of filopodia-associated RSV filaments are indicated with arrowheads. **(B) ARP2 knockdown reduced RSV-induced filopodia.** In samples from two independent experiments similar to the experiment described for A, the number and length of filopodia on siControl- and siARP2-transfected mock-infected RSV-WT-infected cells were evaluated by automated scanning using confocal microscopy. In brief, Z-stacking for Alexafluor_488_ for RSV F protein, DAPI for nuclei, rhodamine phalloidin for F-actin was performed for 50 to 100 different random fields of interest in each coverslip. The length and number of filopodia was measured on 100 cells per treatment per experiment from the surface to the tip of the filopodium. Error bar: SD.

We further visualized the effects of RSV infection and ARP2 knockdown on virus and cell morphology using immunofluorescence confocal microscopy. A549 cells were transfected with siARP2 or siControl for 48 hr, mock-infected or infected with RSV-wild type (RSV-WT) for 24 hr (MOI = 1), fixed, permeabilized, immunostained for RSV F (*green*) and beta-tubulin (*cyan*), and stained with rhodamine phalloidin (*red*) to detect F-actin as a marker for filopodia and with the nuclear stain DAPI (*blue*). All four colors are shown in the images in Fig 7A, and images that individually show the *green*, *cyan*, and *red* channels are shown in S4 Fig. In siControl-transfected RSV-infected cultures (Fig 7A, third row of panels), RSV-infected cells were found to contain long surface projections that were intensely stained with rhodamine phalloidin (*red*), indicative of F-actin content. In addition, these surface projections were deficient in tubulin (*cyan*) (S4 Fig, third row of panels, arrows). The morphology of these surface filaments, and the presence of actin and relative absence of tubulin, was consistent with filopodia, whereas lamellipodia contain abundant tubulin. Filopodia were not prominent in the mock-infected siControl- or siARP2-transfected cells (Fig 7A, top first and second rows of panels), consistent with the previous observations with immuno-SEM, suggesting that they were induced by RSV infection. Moreover, consistent with findings in the immuno-SEM images described above, extensive filamentous extracellular virions were observed in the RSV-infected siControl-transfected cultures (Fig 7A, asterisks indicate free extracellular virions and arrowheads indicate examples of filopodia-associated virions).

The filopodia were reduced in number and length in siARP2-transfected RSV-infected cells (Fig 7A, bottom row; Fig 7B), consistent with the previous observation by immuno-SEM. The abundance of extracellular virions also was markedly reduced. Some variability in the extent of filopodia reduction was observed for the siARP2-transfected RSV-infected cells even within the same wells: in some cells, the cell surface appeared to be completely devoid of filopodia, while in other cells filopodia were present but were reduced in number and were much shorter. One possibility is that this reflects cell-to-cell differences in the efficiency of ARP2 siRNA transfection and knockdown.

We quantified the number and length of filopodia in the siARP2- and siControl-transfected mock-infected or infected cells by automated scanning using confocal microscopy. We counted all filopodia from, on average, 100 cells per treatment per experiment, and the length of each filopodium was measured from the cell surface to the tip of the filopodium. The number and length of filopodia from two independent experiments are summarized in Fig 7B. In the siControl-transfected infected cells, the filopodial length (measured only on the infected cells in the culture, confirmed by RSV-F-staining) was observed to be up to 100 μm, and the longer filopodia were observed in greater numbers in the lower-density cultures, suggesting that length increased with increasing space between cells. In contrast, in siARP2-transfected RSV-infected cells, the number of longer filopodia (measured only on the infected cells, identified by F-actin staining) was greatly reduced (Fig 7B). Compared to RSV-infected cells, fewer filopodia were present on mock-infected siControl and siARP2 cells, and filopodia were less than 20 μm in length. In mock-infected ARP2-knockdown cells, filopodia were furthest reduced in numbers and length. These results confirm that (i) RSV infection is associated with the formation of long filopodia, and (ii) that, filopodia formation involves ARP2.

To confirm these findings and to exclude off-target effects of ARP2 knockdown, we also characterized the role of Wiskott-Aldrich Syndrome protein (WASP), specifically the homolog N-WASP, which has been known to bind directly ARP2/3 complex and stimulate actin polymerization [23]. N-WASP depletion by siRNA caused only a modest reduction in the viability of A549 cells, and was stable over at least 120 hr post-siRNA transfection, and it reduced RSV production in A549 cells (S5 Fig). Importantly, RSV-induced filopodia in A549 cells were reduced or abolished by N-WASP depletion (S6 Fig), confirming that depletion of another factor involved in filopodia formation has a similar effect to that of ARP2 depletion.

### Role for filopodia and ARP2 in RSV spread

**Fig 8:**
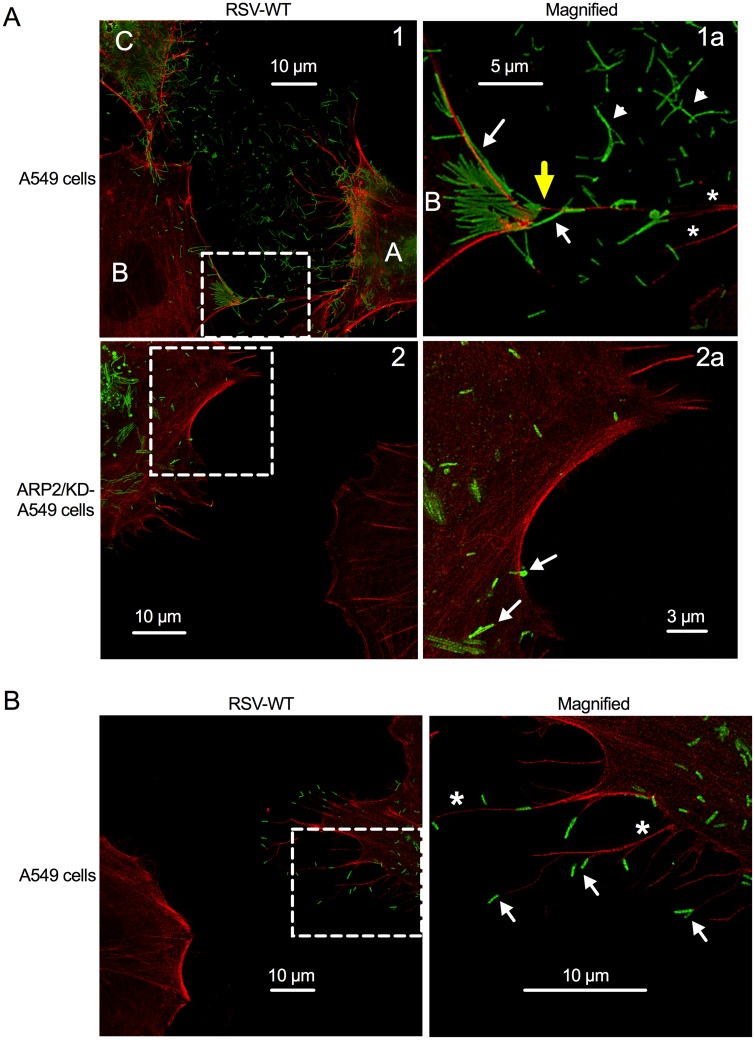
RSV appears to use filopodia for cell-to-cell spread. **(A, B) Stimulated emission depletion** **(STED) imaging of filopodia and virus filaments on RSV-infected cells.** A549 cells or ARP2/KD-A549 cells (i.e., stable ARP2 knockdown) were infected with RSV-WT (MOI = 1). At 24 hpi, cells were fixed and permeabilized. F-actin was visualized by rhodamine phalloidin (*red*); immunostaining for RSV F was done with an RSV F mAb, followed by secondary antibody conjugated with AlexaFluor_647_ (pseudocolored in *green*; part A, top panel) or AlexaFluor_488_ (*green*; part A, bottom panel, and part B). Examples of filopodia are indicated with asterisks, and examples of what appear to be released filamentous RSV particles are indicated with arrowheads, and examples of cell-associated and filopodia-associated RSV are indicated with arrows. The areas in Panels 1 and 2 (left) that are outlined with dashed boxes are enlarged in panels 1a and 2a, respectively (right). Part (A) illustrates that the filopodia of the RSV-infected cells (panel 1, labeled A and C) appear to convey RSV particles to a neighboring cell (labeled B); in the enlargement 1a, a filopodium-cell junction is indicated with a yellow arrow. In panel 2 and enlargement 2a, filopodia-driven cell-to-cell spread was not evident. Part (B) illustrates the RSV virions at the tip of filopodia.

We also examined RSV infection of ARP2/KD-A549 cells versus untransduced A549 cells using stimulated emission depletion (STED) microscopy, which provides higher resolution (Fig 8A and 8B). A549 cells or ARP2/KD-A549 cells (i.e., stable ARP2 knockdown cells) were infected with RSV-WT (MOI = 1), incubated for 24 hr, and fixed, permeabilized, and stained for F-actin (*red*) and RSV F protein (*green*). In A549 cells, we observed abundant extracellular filamentous structures that stained intensely with RSV-F-specific mAb and were consistent with being RSV virions (*green*; examples of free virions are indicated with arrowheads and examples of cell- and filopodia-associated virions are indicated with arrows), suggesting extensive virus shedding (Fig 8A, panel 1a); in contrast, in the infected ARP2/KD-A549 cells, reduced virus shedding was observed (Fig 8A, panel 2a). This result was consistent with the result in the Fig 7A, bottom two rows.

Two A549 cells, labeled “A” and “C” in Fig 8A, panel 1, appear to be infected based on staining for F protein (*green*) and the presence of filopodia (*red*; some examples are indicated with asterisks in panel 1a). Some of the filopodia from cell “A” (Fig 8A, panels 1 and 1a) appear to be contacting (yellow arrow) cell “B” that is located on the left hand side of the panel, and this area of contact in cell B has a number of virus-like particles, whereas the rest of cell B has little or no staining for RSV F (except at the junction of filopodia-driven interaction by cell “C”, shown only in Fig 8A, panel 1) and has minimal filopodia and thus appears to be otherwise uninfected. Thus, the filopodia appeared to be conveying virions from infected cells to uninfected cells. RSV virions were frequently observed at the tip of the filopodia, further supporting the idea of filopodia-driven RSV cell-to-cell spread (Fig 8B). In contrast, filopodia-driven RSV cell-to-cell spread was not apparent in the ARP2/KD-A549 cells (Fig 8A, panels 2 and 2a).

We investigated a possible role for RSV-induced filopodia in viral spread using live cell imaging. A549 cells were transfected with siControl or siARP2, infected with RSV-GFP, and observed with confocal microscopy over time, with images taken every 6 min (MOI = 0.1, imaged from 24 to 48 hpi, S1 and S2 Movies, respectively). The time-lapse images showed that RSV infection resulted in increased cellular motility in the control-knockdown cells, but not in the ARP2-knockdown cells. The control-knockdown infected cells were much more active in migration and showed an increased number of filopodia (visualized as abundant hair-like structures on the surface), compared to the ARP2-knockdown infected cells. As filopodia are a means of cell motility [24], this suggests that the RSV-induced filopodia rendered the RSV-infected cells able to migrate and contact other cells, which appeared to result in the secondary cells becoming GFP-positive. This was inhibited by ARP2 knockdown.

In order to visualize actin in live cell experiments, we generated a Red F-actin-A549 cell line, which stably expresses a 17-amino-acid-long actin-binding domain linked genetically to red fluorescence protein (RFP). This fluorescent fusion protein binds to F-actin, reportedly without interfering with its function [25]. siControl- and siARP2-transfected cells were mock-infected (S3 and S4 Movies, respectively) or infected with RSV-GFP (MOI = 0.1, imaged from 24 hpi to 48 hpi, S5 and S6 Movies, respectively). In response to RSV-GFP infection, the control-knockdown cells formed filopodia containing red actin, and were motile and appeared to spread infection to neighboring uninfected cells. A region of interest is magnified from RSV-GFP infected Red F-actin A549 cell line (MOI = 0.01, imaged from 24 to 48 hpi) to show viral cell-to-cell spread (S7 Movie).

### ARP2 depletion reduced the progression of RSV infection

To more closely mimic RSV infection in non-ciliated respiratory epithelial cells of the lower respiratory tract, we also performed these experiments using confluent monolayers of Red F-actin-A549 cells (S8, S9, S10, and S11 Movies). Time lapse imaging of siControl transfected Red F-actin-A549 cells confirmed that RSV-GFP infected cells (S10 Movie) were more dynamic than uninfected cells (S8 Movie). Interestingly, the infected cell monolayers were disrupted over time by the cytopathic effect of RSV-GFP; any void was filled quickly by actively migrating newly RSV infected (newly green) cells, resulting in efficient spread of RSV infection through the monolayer (S10 Movie). As observed previously in non-confluent monolayers, ARP2 knockdown reduced the motility and filopodia formation of infected cells (S11 Movie). As a consequence, the cell monolayers were retained longer in siARP2 transfected cells (S11 Movie) than in siControl cells (S10 Movie).

**Fig 9:**
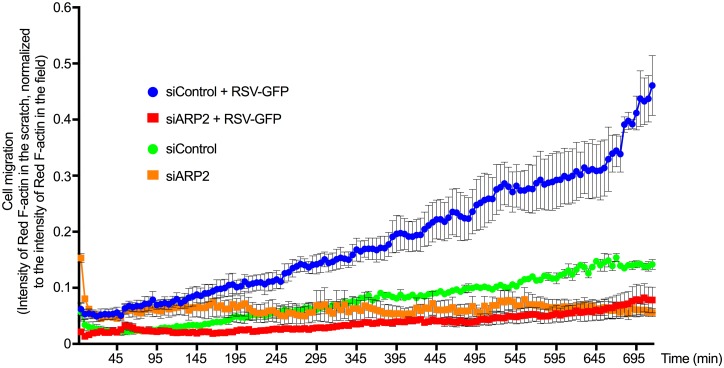
ARP2 knockdown reduced RSV-induced cell migration. Red F-actin-A549 cells were transfected with siARP2 or siControl. 48 hr after transfection, cells were mock-infected or infected with RSV-GFP (MOI = 1). At 24 hpi cell monolayers were scratched with a pipette tip followed by imaging every 5 min for 12 hr. Cell migration was measured by quantifying the intensity of Red F-actin in the scratch normalized to the intensity of Red F-actin in the field at each time point. Data obtained from three replicates of each sample. Error bar: standard error of mean.

We also used a scratch-wound assay based on A549 cells as a surrogate model for tissue damage caused by the cytopathic effect of RSV, and we measured the migration of RSV infected cells in this assay (Fig 9). Confluent siControl- or siARP2-transfected Red F-actin-A549 cell monolayers were mock-infected or infected with RSV-GFP (MOI = 1). At 24 hpi, the monolayers were scratched with a pipette and were followed by imaging every 5 min from 24 hpi to 36 hpi. To measure the migration of Red F-actin-A549 cells into the wound area, we scored the red intensity in the scratched area over time. We confirmed that RSV infection considerably increased cell motility, whereas ARP2 depletion drastically reduced RSV-induced motility (Fig 9). In vivo, RSV promotes shedding and cell death of bronchial and lung epithelial cells [26,27]. This suggests that the induction of ARP2-dependent mobility of RSV infected cells promotes the spread of RSV infection and cytopathogenicity in the respiratory tract.

### The induction of filopodia and cell motility was much more robust with RSV than with HPIV3 or HMPV

**Fig 10:**
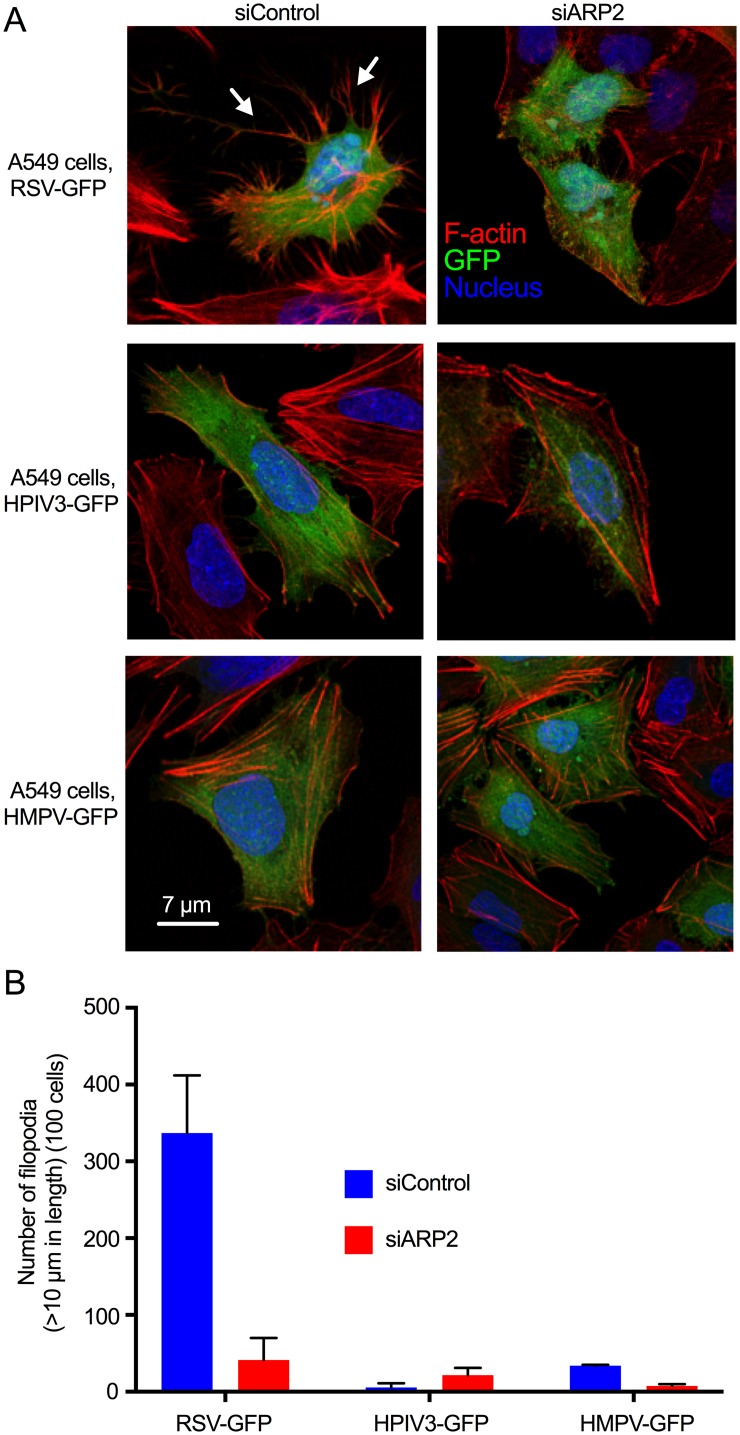
RSV induced filopodia more robustly than HPIV3 or HMPV infection. **(A)** A549 cells were transfected with siARP2 or siControl. 48 hr after transfection, cells were infected with RSV-GFP, HPIV3-GFP or HMPV-GFP at MOI = 1. At 24 hpi, cells were fixed and permeabilized, and F-actin was stained with rhodamine phalloidin. Nuclei were stained with DAPI. Infected cells were detected by GFP expression. RSV-induced filopodia are indicated with arrows. **(B)** The number and length of filopodia were evaluated by automated scanning using confocal microscopy. In brief, Z-stacking for GFP for virus, DAPI for nuclei, rhodamine phalloidin for F-actin was performed for 50 to 100 different random fields of interest in each coverslip. The length and number of filopodia was measured on 100 cells per treatment per experiment from the surface to the tip of the filopodium. Data were combined from two independent experiments. Error bar: SD.

We compared the efficiency of induction of filopodia by RSV, and their effects on motility and virus spread, to that of two other common respiratory paramyxoviruses, namely HPIV3 and human metapneumovirus (HMPV). A549 cells were transfected with siARP2 or siControl; infected with RSV-GFP, HPIV3-GFP, and HMPV-GFP; incubated for 24 hr; fixed, permeabilized, stained with rhodamine phalloidin (*red*) and DAPI (*blue*); and examined by confocal microscopy. Examination of infected cells (*green*) showed that RSV-GFP induced abundant filopodia (Fig 10A, top row left panel; examples of filopodia are indicated with arrows), as expected. In contrast, HPIV3-GFP and HMPV-GFP induced few filopodia-like structures on the infected cell surface (Fig 10A, middle and bottom rows, respectively). Long intracellular actin filaments were observed in each of the cultures, and were especially evident in uninfected cells, but the induction of filopodia was much more robust in response to RSV (Fig 10B). As expected, RSV-induced filopodia formation was greatly reduced in siARP2-transfected, RSV-GFP-infected cells (Fig 10A, top right), but ARP2 knockdown had little effect on the appearance of HPIV3-GFP and HMPV-GFP infected A549 cells (Fig 10A, right middle and bottom panels).

The ability of HPIV3-GFP and HMPV-GFP to induce mobility was investigated in parallel with RSV-GFP using Red F-actin-A549 cells and live cell imaging. Infection with RSV-GFP induced motility and appeared to facilitate viral spread to neighboring uninfected cells (S12 Movie), whereas this was not observed with HPIV3-GFP (S13 Movie), and was minimal with HMPV-GFP (S14 Movie).

### The RSV F protein expression induced filopodia-like structures

**Fig 11:**
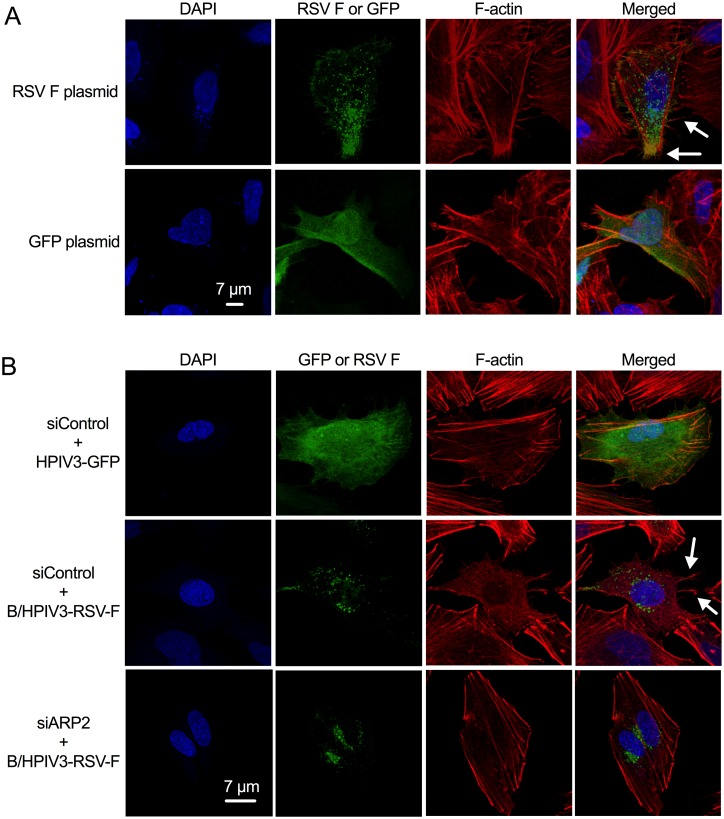
Expression of RSV F is sufficient to induce RSV-associated filopodia. **(A) RSV F expression from transfected plasmid.** A549 cells were transfected with a eukaryotic expression plasmid expressing RSV F or GFP. 12 hr post-transfection, cells were fixed and permeabilized. RSV F was detected with an RSV F mAb followed by anti-mouse AlexaFluor_488_ secondary Ab (*green*). F-actin was stained with rhodamine phalloidin (*red)*, and nuclei were stained with DAPI (*blue*). Examples of filopodia are indicated with arrows. **(B) RSV F expression from a viral vector.** A549 cells were transfected with siARP2 or siControl. 48 hr after transfection, cells were infected with B/HPIV3-RSV-F or HPIV3 (MOI = 1). The cells were fixed at 24 hpi and permeabilized, and F-actin was stained with rhodamine phalloidin (*red*), and nuclei were stained with DAPI (*blue*). Examples of filopodia are indicated with arrows.

Since the RSV F protein mediates viral fusion and thus has dramatic effects on the cell plasma membrane, we investigated whether it induces filopodia formation. We expressed RSV F from a eukaryotic expression plasmid transfected into A549 cells, in parallel with a control plasmid expressing GFP. At 12 hr post-transfection, the cells were fixed, permeabilized, and stained with DAPI, rhodamine phalloidin and mAb specific for the RSV F protein, and analyzed by confocal microscopy (Fig 11A). Long intracellular actin filaments were observed in each of the cultures, but the induction of filopodia-like structures on the transfected cells (Fig 11A, arrows) was observed in response to RSV F but not GFP expression.

We also investigated the effect of the expression of RSV F protein on filopodia formation using a chimeric bovine/human (B/H) PIV3 (B/HPIV-3) that consists of BPIV3 in which the F and HN genes have been replaced by those of HPIV3, and which in addition expresses RSV F from an added gene [28]. A549 cells were transfected with siARP2 or siControl followed by infection with B/HPIV3-RSV-F (Fig 11B). HPIV3-GFP infected cells, included here for comparison (Fig 11B, top row of panels), had minimal induction of filopodia, as already shown in Fig 10. In contrast, the expression of the RSV F protein from B/HPIV3-RSV-F in control-knockdown cells induced filopodia-like structures on the infected cells (Fig 11B, middle panel, arrows). This was largely blocked by ARP2 knockdown (Fig 11B, bottom panel).

## Discussion

Actin is a major component of the cytoskeleton, and cell shape, motility, and a multitude of dynamic intra- and intercellular processes are dependent on actin rearrangement [9]. Actin-dependent cellular functions require precise regulation of actin polymerization. The ARP2/3 complex plays an important role in the initiation of F-actin polymerization (also called nucleation) during diverse cellular processes [29]. The ARP2/3 complex is one of the three major classes of factors for actin nucleation [together with formins and the tandem-monomer-binding family] [21,30]. Viral infection can modulate the actin cytoskeleton morphology, reviewed in [10]. Many enveloped and nonenveloped viruses interact with the actin cytoskeleton during virus entry [10]; actin involvement in RSV endocytosis or macropinocytosis has been described previously [5,31]. While this manuscript was in preparation, it was shown that ARP2/3 complex-dependent actin rearrangement is required for alphavirus trafficking and egress at late time points after infection [32]. Actin is also involved in RSV replication [13], gene expression, and morphogenesis [11,12,33]. However, the mechanisms for actin involvement in RSV infection are poorly understood, and no information was available on the role of ARP proteins during RSV infection. We identified ARP2 in a comprehensive genome-wide siRNA screen targeting approximately 21,500 human genes, performed on RSV-infected human lung epithelial A549 cells. In the present study, we have systematically investigated the effect of ARP2 on the RSV replicative cycle.

For several other viruses, actin polymerization has been shown to play a role in viral entry, and different viruses use the cellular actin system differently. For example, primate lentiviruses enter cells by membrane fusion followed by initiating ARP2/3 complex-driven actin polymerization to generate a mechanical force that allows the lentiviral core complex to pass through the cortical layer and migrate to nucleus. Even though vaccinia viruses enter cells by membrane fusion at the cell surface, cell cytoskeletal rearrangement immediately after viral attachment is required for viral entry and transport through the cytoplasm [34]. One route of RSV entry seems to be through Rab5-positive, fluid-filled macropinosomes. Specifically, RSV binding at the cell surface activates a signaling cascade for actin rearrangement, resulting in viral entry through macropinocytosis [5]. Therefore, we began the present study by investigating possible effects of APR2 on RSV entry and early steps in RSV replication.

Examination of the kinetics and distribution of viral gene expression by flow cytometry showed that ARP2 knockdown had no or only minor effects on the efficiency of infection and viral gene expression during the first 24 hr following inoculation (which approximately spans the time from the initiation of infection to the time when virus production has become robust) but thereafter ARP2 knockdown reduced the number of infected A549 cells without affecting the magnitude of gene expression per cell. We also analyzed RSV mRNA and protein expression over time by qRT-PCR and Western blotting, and similarly showed little or no effect prior to 24 hpi, and an overall reduction in gene expression after 24 hpi. A second ARP2-specific siRNA (Hs_ACTR2_7, QIAGEN) was evaluated with similar results, although the efficiency of knockdown was marginally lower.

We also investigated whether the ARP2/3 complex is involved in early steps of RSV infection by using the potent ARP2/3 complex inhibitor CK-666 [17]. We found that this inhibitor did not reduce RSV entry at concentrations that had previously been shown to be effective in inhibiting ARP2/3 complex-driven actin nucleation [18], while the macropinocytosis inhibitor EIPA did reduce RSV entry, as previously shown [5]. Thus, the ARP2/3 complex did not seem to be essential for any steps of RSV entry or early events in the viral replicative cycle, in particular for actin nucleation during macropinocytosis of RSV. However, actin nucleating factors other than the ARP2/3 complex could contribute on actin nucleation for the more localized macropinosome formation during RSV entry. Even though macropinocytosis is a transient actin dependent endocytic process, it is primarily associated with cell-wide plasma membrane ruffling, and involves formation of large vacuoles for non-selective internalization of fluid and membranes [35,36]. The Ras superfamily of GTPases plays an important role in regulating ruffle formation for macropinocytosis [37], whereas other Rho GTPases such as cell division cycle 42 (Cdc42) regulates ARP2/3 complex-driven actin polymerization for filopodia formation [38,39]. There is extensive overlap and crosstalk, and dynamic interactions are evident among Rho GTPases signaling pathways [40,41]. A coordinated activation of several Rho GTPases was suggested to be involved in the cytoskeleton rearrangement induced by HMPV for its cell-to-cell spread [42]. Further research is necessary to understand whether RSV modulates the signaling of different GTPases for cytoskeleton reorganization during entry.

We next investigated a possible role of ARP2 in RSV production, syncytium formation, budding, and virion morphology. ARP2 knockdown resulted in a 10-fold reduction in RSV release into the supernatant of infected A549 cells when measured two and three days post-infection, while the release of HPIV3 into the supernatant was only minimally reduced in siARP2-transfected cells. A similar reduction in RSV production was observed in another respiratory cell line, Calu-3 (S1 Fig) when infected with RSV-WT (MOI = 1). This reduction in RSV production was partly evident by 24 hpi, suggesting that it reflected, at least in part, reduced virus yield per infected cell. The reduction was greater by 48 and 72 hpi, suggesting that it also reflected reduced secondary infection. We did not observe substantial effects of ARP2 depletion on RSV fusion, suggesting that this was one aspect of RSV spread that was not affected by ARP2 knockdown.

Consistent with the reduction in RSV production, we also observed reduced extracellular RSV particles on the cell surface by microscopy. TEM and immuno-SEM showed that there were many fewer virus-like particles and filaments on the surfaces of ARP2-knockdown infected cells compared with control-knockdown infected cells. The TEM studies suggested that, while fewer RSV progeny particles were present, their general morphology appeared unchanged compared to control-knockdown infected cells. However, analysis by immuno-SEM showed that, while the general morphology of progeny virions on the surface of ARP2-knockdown infected cells was similar to that of control-knockdown infected cells, their arrangement appeared to be less orderly. A contribution of actin filaments to RSV virion maturation and egress, and to the formation of viral filaments, has been reported previously [6,11,12,43,44]. A role of actin and myosin in the transport of the viral ribonucleoprotein (RNP) complex has also been described previously, and the role of the ARP2/3 complex in the terminal stage of virus budding and in the process of pinching off from the cell surface has been proposed [6]. Conversely, a previous study showed that F protein trafficking to the cell surface and assembly of RSV filaments appeared to take place even when cytoskeleton rearrangement is blocked by actin inhibitors [45]. Our results suggest that efficient RSV budding indeed depends on functional ARP2/3 nucleation and actin rearrangement.

Perhaps our most interesting observation was that RSV infection in A549 cells induced filopodia, which are finger-like membrane protrusions that are rich in F-actin and mostly deficient in tubulin. Lamellipodia (which are larger, broad, flat protrusions containing branched actin and tubulin) also appeared to be formed in response to RSV infection, although this was not further investigated in the present study. Filopodium formation is a highly orchestrated multistep process associated with actin cytoskeleton rearrangement; however, the underlying mechanism for initiation and maintenance of filopodia has not been fully characterized [46]. Compared to RSV viral filaments, filopodia are larger in diameter and largely devoid of RSV proteins. We discriminated filopodia from lamellipodia based on the former’s thin extended morphology, low microtubulin content, and intensity of F-actin staining [47]. Membrane protrusions have been shown to serve as vehicles for pathogen spread for *Listeria monocytogenes* and *Shigella flexneri*. These bacteria induce actin motility when they enter into the cytosol, which facilitates bacterial interaction with the plasma membrane. Bacteria have been observed to be transported within cell membrane protrusions that extended into invaginations in adjoining cells, contributing to cell-to-cell spread, reviewed in [48]. In the present study, STED observations of RSV particles on A549 cells suggested that clustered filamentous RSV virions are present on filopodia, similar to filopodia-associated virions which were observed on cells infected with murine leukemia virus (MLV) [49] and African swine fever virus (ASFV) [50]. We also detected filamentous RSV virions clustered at the tips of filopodia, suggesting that mature virion particles are shuttled to neighboring uninfected cells. In the STED observations, immunostaining was done on permeabilized cells. This technique does not discriminate whether mature virions were located intracellularly or extracellularly on the plasma membrane. However, immuno-SEM performed using the same Ab in non-permeabilized infected cells showed that mature RSV virions are present extracellularly on the plasma membrane of filopodia, as might have been expected. These results suggest that RSV is shuttled on the exterior of the filopodia to infect neighboring cells. However, RSV potentially might also shuttle through filopodia using intracellular vesicle transport, which is currently under investigation.

We found that expression of the RSV F protein from a plasmid vector or a heterologous viral vector was sufficient to induce filopodia, although the number and length of these structures were reduced compared to RSV-infected A549 cells. Our study identifies a new mechanism by which the F protein promotes RSV spread, namely thorough inducing filopodia formation, thus promoting filopodial spread of RSV. Filopodia induction in A549 cells due to the expression of RSV F from a vector also was sensitive to ARP2 depletion, similar to the filopodia induced by RSV infection. RSV-mediated filopodia induction was not observed in either Calu-3 or Vero cells, despite the evidence that ARP2 contributes to RSV production in both cell types (Calu-3 cells, S1 Fig). Further research is necessary to understand whether cell-type specificity contributes to RSV-driven filopodia induction, whether the RSV F protein uses the actin network and filopodia for intra-and/or inter-cellular transport, whether F expression activates filopodia signaling pathways, and whether it acts as a nucleation promoting factor (NPF) for ARP2/3 complex-driven actin polymerization.

We found that in addition to filopodia formation, RSV infection increased cellular motility, which promoted virus spread to neighboring cells. Virus-induced cell migration has been reported for vaccinia virus [51]. Live cell imaging in the infected Red-F-actin A549 cells illustrated that filopodia-driven RSV spread was facilitated by cell mobility, and ARP2 knockdown reduced RSV spread not only by inhibiting the induction of filopodia, but also by restricting cell mobility.

Filopodia-driven cell-to-cell spread was much more robust for RSV compared to HPIV3 and HMPV. A recent study showed that HMPV induces intercellular extensions of a branched actin network for its spread in human bronchial epithelial cells. However, a branched actin network was less obvious in A549 and Vero cells [42]. Our study shows that RSV exploits the actin cytoskeletal system of human lung epithelial cells for its spread. If our findings are relevant to the situation *in vivo*, they suggest that RSV may promote cell motility, in particular in areas subject to RSV cytopathic effect. Our findings also suggest that RSV induces filopodia (through the F protein), and that filopodial spread allows rapid dissemination of the virus to new target cells. Filopodia may also facilitate spread in the presence of biophysical barriers such as mucous layers on mucosal surfaces [52,53], and spread under these conditions may reduce exposure to virus-neutralizing antibodies [53,54]. In future studies, we will further investigate the possibility of virus-induced filopodia formation and filopodia-driven viral spread in primary human airway epithelial cells.

Filopodial protrusions can function as sensors of the local environment in migrating cells, and the spatial-temporal dynamics of filopodia have been described [55]. The ARP2/3 complex directly contributes to filopodia formation by nucleating actin filaments in lamellipodia [56], but the ARP2/3 complex by itself has little actin-nucleating activity. NPF binding to the ARP2/3 complex activate actin-polymerization and dictate where and when nucleation originates in the cell [8]. Indeed, we showed that a depletion of another potent NPF, N-WASP, reduced or abolished the formation of RSV-induced filopodia, and consequently reduced RSV production in A549 cells. Results of the N-WASP depletion studies confirmed that RSV modulates filopodia signaling for its direct cell-to-cell spread. However, NPF activities are highly regulated by signal-transduction pathways such as Rho-family GTPases, Cdc42 and Rac (reviewed in [29]). A contribution of the small GTPase RhoA, which acts through myosin II, and affects motility independently of the ARP2/3 complex, in RSV infection has already been reported [57]; future studies are required to explore the contributions of other small GTPases Rac1 and Cdc42 to filopodia-driven RSV spread. More work is needed to evaluate if the RSV F protein, and possibly other viral proteins, interact with ARP2/3 complex-specific NPFs to initiate ARP2/3 complex-driven actin polymerization.

In conclusion, ARP2 knockdown reduced RSV production, primarily by reducing viral budding and spread. There was no evidence of effects on RSV entry, or of significant effects on events in the viral replicative cycle prior to approximately 24 hpi, a time when the production of progeny virions and virus spread becomes robust. Our results show that RSV infection increases cell motility, induces filopodia formation, that filopodia promote RSV spread, and that this occurred in human lung epithelial A549 cells. ARP2, which is part of ARP2/3 complex, was shown to be necessary for induction of filopodia in RSV-infected cells, for increased motility of RSV-infected cells, and for filopodia-mediated spread of RSV. Filopodia formation and particle formation in A549 cells were also dependent on N-WASP, which binds to ARP2/3 and stimulates actin polymerization. ARP2 depletion reduced the spread of RSV infection in confluent monolayers *in vitro*. If these results translate to respiratory epithelium of the lower respiratory tract, this would suggest that inhibition of actin polymerization may reduce the spread of RSV infection in the lower respiratory tract. This identifies a previously unappreciated effect of the RSV F protein, a previously unappreciated mechanism of RSV spread, a novel cellular factor for RSV spread, a new insights on the effect of RSV infection on cellular functions, and a potential therapeutic target to combat RSV infection.

## Materials and Methods

### Cells and viruses

Calu-3 (ATCC HTB-55) and A549 (ATCC CCL-185) cells were obtained from the ATCC. Calu-3 cells were maintained in EMEM (ATCC), supplemented with 10% fetal bovine serum (FBS) (Life Technologies). A549 cells were maintained in F-12 complete medium [F-12 Nutrient Mixture (Life Technologies), supplemented with 10% FBS and 1% L-glutamine]. Recombinant RSV expressing enhanced GFP from an added gene, inserted between the P and M genes (RSV-GFP), was described previously [14], and is a derivative of recombinant RSV-WT (A2 strain). For all experiments, RSV was grown in Vero cells and purified by centrifugation and banding in discontinuous 30% to 60% (wt/vol) sucrose gradients as previously described [58]. Similarly, HPIV3-GFP [59], HMPV-GFP [60], and B/HPIV3-RSV-F were sucrose-purified. B/HPIV3-RSV-F is a chimeric B/HPIV3 based on BPIV3 in which the fusion (F) and hemagglutinin-neuraminidase (HN) surface glycoproteins have been replaced by their counterparts from HPIV3, and which in addition expresses a codon-optimized version of the RSV F protein from an added gene [28].

Viral infections were done at an MOI of 1 unless specified otherwise. A549 cells were seeded in 24-well plates and were incubated with virus in 100 μl of F-12 medium for 1 hr, and rinsed twice with F-12 medium. Infected cells were incubated in F-12 medium with 2% FBS and 1% L-glutamine. For viral infections of Calu-3 cells, EMEM medium was used instead of F-12 medium. For live cell imaging, infected cells were kept in media with 25 mM HEPES (Life Technologies).

### Stable cell lines

ARP2/KD-A549 cell line was generated by using a lentiviral vector-based construct expressing three target-specific 19–25 nt (plus hairpin) small hairpin RNAs designed to knockdown gene expression (sc-29737-V, Santa Cruz Biotechnologies, Inc) according to the manufacturer's recommendations with minor modifications. Briefly, 1x10^5^ A549 cells were infected at a range of MOIs from 0.1 to 0.01 with 6 μg/ml polybrene (Santa Cruz Biotechnologies, Inc) and incubated in F-12 complete medium with 10 μg/ml puromycine (Takara Clontech). A clonal population was obtained from a single cell. Similarly, we generated Red F-actin-A549 cells that stably express Red F-actin, which is a fusion protein that combines an actin-binding domain with a red fluorescent protein (RFP) (Ibidi). This was created using the lentiviral vector-based construct rLV^Ubi^-LifeAct-TagRFP (Ibidi), and clonal population was obtained.

### siRNA transfection

siRNA transfections were done in 24-well plates using a reverse transfection protocol (i.e., cells in suspension were added to wells containing the siRNA and transfection reagent), which was optimized using the KDalert GAPDH Assay Kit (Life Technologies). Briefly, the knockdown efficiency of different concentrations of siRNA (siGAPDH) and RNAimax transfection reagent (Life Technologies) was tested on 3x10^4^ cells. GAPDH knockdown was determined by measuring the enzymatic activity according to the manufacturer’s instructions. Transfection with 1μl of RNAiMax transfection reagent and 7.5 μl of siRNA (2 μM siRNA concentration) reduced GAPDH activity by more than 80% without compromising cell viability. Therefore, this concentration was used for protein knockdown experiments in A549 cells unless otherwise mentioned. In Calu-3 cells, protein knockdown was done similarly by using twice the amount of siRNA on 100,000 cells. Reverse transfections of A549 or Calu-3 cells were performed 48 hr before virus infection and siARP2 (s223082, Life Technologies) and siControl (Silencer Select Negative Control #2, Life Technologies) were used for all ARP2 knockdown experiments, unless otherwise specified. For N-WASP knockdown in A549 cells, siN-WASP (137397, Life Technologies) was used.

Cell viability was evaluated using resazurin (alamarBlue, Life Technologies) according to the manufacturer's protocol. Briefly, a 10% volume of alamarBlue was added to the cell culture media and incubated at 37°C for 3–4 hr. To evaluate the cell viability, alamarBlue fluorescence, a marker for metabolic activity, was analyzed using a Synergy 2 Multi Mode microplate reader (BioTeK).

### Western blot analysis

Ten micrograms of total protein was separated on 4–12% Bis-tris SDS polyacrylamide gels, followed by dry blot transfer onto polyvinylidene floride (PVDF) membranes according to the manufacturer’s instructions (Life Technologies). For viral protein detection, samples were denatured at 90°C for 10 min with 1% reducing agent before gel electrophoresis. The PVDF membranes were incubated in LI-COR blocking buffer (1:1 in PBS) (LI-COR Biosciences) for 1 hr, followed by overnight incubation with primary Ab in blocking buffer. The membranes were washed 4x for 5 min each in wash buffer (PBS with 0.1% Tween 20, Sigma-Aldrich), followed by incubation with secondary IRDye Ab (LI-COR Biosciences) for 1 hr. After washing 4x for 5 min in wash buffer, fluorescence was analyzed using the Odessey imaging system (LI-COR Biosciences). ARP2 was detected using a rabbit mAb (ab129018, Abcam) and a goat anti-rabbit IRDye800 Ab (LI-COR Biosciences). Alpha-tubulin was detected using a mouse mAb (T6199, Sigma-Aldrich) and a goat anti-mouse IRDye680 (LI-COR Biosciences). N-WASP was detected using a rabbit mAb (30D10, Cell Signaling Technologies) and a goat anti-rabbit IRDye800 Ab. RSV F and P were detected using mouse mAbs (ab43812 and ab94965, respectively) for primary and respective secondary Abs (goat anti-mouse IRDye800 and IRDye680, respectively). GAPDH was detected using a primary rabbit pAb (sc25778, Santa Cruz Biotechnologies, Inc.) and a goat anti-rabbit IRDye680 Ab.

### Quantitative (q) RT-PCR

Cells were harvested and intracellular RNA was purified by RNAeasy (QIAGEN). The RNA concentration was determined, and 100 ng of RNA was used for first-strand cDNA synthesis (Superscript III, Life Technologies) using oligo(dT) primers (Life Technologies). qRT-PCR was performed using TaqMan assays (ARP2: Hs00855199, Life Technologies and RSV ORFs: primer sequence available upon request) in the 7900HT Fast Real-Time PCR System (Life Technologies), and fold changes were calculated using the delta-delta-CT method using 18S RNA (Hs99999901, Life Technologies) or beta-actin (Hs99999903, Life Technologies) as a calibrator.

### Virus titration

For quantitation of free RSV, the cell culture medium was harvested without disturbing the cells and then clarified at 300 x g for 10 min. For quantification of total RSV in the culture (cell-associated and free virus), monolayers were scraped into the cell culture medium, collected and vortexed vigorously three times for ten seconds each, and clarified by centrifugation at 300 x g for 5 min. Supernatants were snap frozen on dry-ice and kept at -80°C. RSV titration was done by plaque assay on Vero cells in which viral plaques were visualized by immunostaining with three mAbs against RSV F [61]. Alternatively, in the case of RSV-GFP and HPIV3-GFP, plaques were visualized directly by GFP expression using a Typhoon Trio+ imager (GE Healthcare), followed by quantification using the software ImageJ.

### Analysis of protein expression by flow cytometry

Cells were washed in PBS and detached with 0.5% TrypLE Select 1x (Life Technologies), incubated with FBS to neutralize trypsin activity, pelleted by centrifugation at 300 x g for 5 min, and incubated with Live/Dead near-infrared fluorescence reactive dye (Life Technologies) for 30 min. Cells were washed and fixed and permeabilized with BD Cytofix/Cytoperm buffer (BD Biosciences). Multicolor flow cytometry was used to analyze the expression of the RSV F and M2-1 proteins and GFP simultaneously in infected cells. To detect RSV F, we used a commercial biotin-labeled mAb (133-1H MAB8262B-5RSV, Millipore), and Strepatvidin BV605 (Biolegend). For RSV M2-1, we labeled an M2-1 specific mAb (RSV5H5, Abcam) with AlexaFluor_647_ or Site-Click R-PE antibody labeling kit (Life Technologies) according to the manufacturer’s instruction. Single color antibody labeled cells for each antibody were used for compensation, and fluorescence minus one controls were included to aid in setting gates. Acquisition was done until 20,000 live single cells were recorded and cells were gated on live singlet cells, followed by gating on GFP, F and M2-1 positive cells using a BD LSR Fortessa flow cytometer and FACSDiva software (BD Biosciences), followed by analysis with FlowJo software version 9.7.2 (FLOWJO, LLC).

### Entry assays

5x10^5^ cells per well in 6-well plates were treated for 1 hr with a final concentration of 100μM of the entry inhibitors EIPA or CK-666 (Sigma-Aldrich) [17,18] for 1 hr. Optimal, non-toxic concentrations were determined in preliminary experiments. The inhibitors were made as concentrated stock solutions in DMSO, and the final DMSO concentrations in the cultures were 1% or less, and a DMSO (solvent-only) control was included. Following the 1 hr incubation, cells were infected with RSV-GFP at an MOI of 5, or with a comparable amount of UV-inactivated RSV-GFP for 6 or 12 hr, or was incubated with dextran fluorescein (Life Technologies) for 30 min. Plates were transferred onto wet ice to stop entry. To quantify dextran or RSV internalization by flow cytometry, cells were detached, incubated with Live/Dead near-infrared fluorescence reactive dye, and fixed with BD Cytofix/Cytoperm buffer. Dextran fluorescein or GFP single live positive cells were quantified using the FACS CANTO II (BD Biosciences). FACS data analysis was done using FlowJo software version 9.7.2. UV-inactivated RSV-GFP was prepared using a Stratalinker UV cross-linker (Agilent) at 0.5 J/cm^2^. Complete inactivation was evaluated by plaque assay as described previously [62].

### Syncytia quantification

Infected cells grown on coverslips were fixed, permeabilized, and stained with DAPI and rhodamine phalloidin as described for confocal microscopy, and images for quantification were acquired using a Leica DMI6000 inverted wide-field microscope equipped with DAPI, GFP, and rhodamine filter cubes, a HCX Pl Fluotar 20X/0.4 objective, and a DFC 360FX monochrome camera. The entire coverslip was imaged in a single plane using the tiling and predictive focus features in LAS software. Using Imaris image processing software, large regions of the tiled image were selected; nuclei were automatically counted using the “Spot” feature, while syncytia and infected cells were counted manually. Per coverslip, at least 5000 cells were analyzed. Syncytia were counted when they contained ≥3 nuclei and were GFP-positive (which was the case for the vast majority of syncytia).

### Electron microscopy

For TEM, A549 cells were seeded on Thermanox coverslips (Electron Microscopy Sciences) at a density of 3x10^4^ cells per well and reverse transfected with siRNA for 48 hr, followed by infection with RSV-GFP at an MOI of 1 for 24 hr. Cells were fixed with 2.5% glutaraldehyde in Sorensen’s phosphate buffer (Electron Microscopy Sciences). Samples were post-fixed 1 hr with 0.5% osmium tetroxide/0.8% potassium ferricyanide, 1 hr with 1% tannic acid and overnight with 1% uranyl acetate at 4C. Samples were dehydrated with a graded ethanol series, and embedded in Spurr’s resin. Thin sections were cut with a Leica UCT ultramicrotome (Vienna) stained with 1% uranyl acetate and Reynold’s lead citrate prior to viewing at 120 kV on a FEI Tecnai BT Spirit transmission electron microscope (Hillsboro, OR). Digital images were acquired with an AMT digital camera system (AMT) and processed using Adobe Photoshop CS5 (Adobe Systems Inc).

For immuno-SEM, A549 cells were seeded on silicon chips (Ted Pella Inc.) at a density of 3x10^4^ cells per well were first reverse transfected with siRNA for 48 hr, followed by infection with either RSV-GFP at an MOI of 5 for 24 hr. Cells were fixed with 4% PFA in PBS for 30 min, blocked with 3% bovine serum albumin (BSA) in PBS, incubated with mAb (ab43812) (1:100 dilution in 0.1% BSA) overnight at 4°C, washed with 3% BSA in PBS, followed by incubation with secondary Ab (goat anti-mouse conjugated with 15nm colloidal gold particle) (EM.GMHL15, BBInternational). After immuno-labeling, the specimens were fixed with 2.5% glutaraldehyde in Sorensen’s phosphate buffer overnight at 4C, post-fixed for 1 hr with 1% osmium tetroxide, and dehydrated in a graded ethanol series. The samples were critical-point dried under CO_2_ in a Bal-Tec model cpd 030 dryer (Balzers), mounted on aluminum studs, and sputter coated with 35 angstroms of chromium in a model IBS/TM200S ion beam sputterer (South Bay Technologies). Specimens were viewed at 10 kV in a Hitachi SU-8000 field emission SEM (Hitachi) using mixed backscatter and secondary imaging modes.

### Confocal microscopy

A549 cells were seeded onto cover glasses (Deckglaser) at a density of 3x10^4^ cells per well and reverse transfected with siRNA for 48 hr, followed by infection with RSV-WT at an MOI of 1. At 24 hpi, cells were washed with PBS, fixed with 4% PFA (Polysciences, Inc.) in PBS for 10 min at room temp, permeabilized with 0.5% Triton-X100 (Sigma-Aldrich) for 10 min and then blocked with 3% BSA solution in PBS for 2 hr. Cells were then incubated with primary Abs (mouse mAb, ab43812 for RSV F protein and rabbit mAb, 9F3, Cell Signaling Technology, Inc.) (diluted 1:500 and 1:100, respectively in 0.1% BSA solution) overnight at 4°C, followed by incubation with secondary Abs (anti-mouse AlexaFlour_488_ or anti-rabbit AlexaFlour_647_) (diluted 1:200 in 0.1% BSA solution) for 2 hr at 4°C, followed by incubation with rhodamine phalloidin (diluted 1:500) (Cytoskeleton Inc) for 30 min at room temp in the dark, followed by nuclear staining with NucBlue Fixed cell Stain ReadyProbes (Life Technologies) for 20 min in the dark. Coverslips were washed with PBS before mounting on microscope slides (Scientific Device Laboratory) using ProLong Gold anti-fade mounting media (Life Technologies). Confocal images were collected using a Leica SP8 confocal microscope (Leica Microsystems) enabled with 63X/1.4NA and 40X/1.25NA oil immersion objectives and hybrid (HyD) detectors. To visualize the details of finer structures such as fiolopodia and lamellipodia, Z stack slices of 0.3 to 0.5 μm were collected and random fields of the cover slip were acquired with automated tiling methods to get an unbiased data set of approximately 50 to 100 random fields of interest. Some confocal images were subsequently deconvolved using Huygens software (Scientific volume imaging) to improve resolution. Filopodial structures were discriminated from lamellipodia using microtubulin Ab staining (absent in filopodia), and number of filopodia and their length was quantified using the “Measurepoint” module in Imaris image analysis software. Data was averaged from two independent experiments.

### STED imaging

RSV-WT infected A549 or ARP2/KD-A549 cells were seeded onto coverslips and fixed and permeabilized at 24 hpi as described above. Cells were stained with a mAb specific for RSV-F and secondary goat anti-mouse AlexaFlour_488_ or AlexaFlour_647_. F-actin was stained with rhodamine phalloidin. Images were collected on a Leica TCS SP8 STED 3X system equipped with a white light excitation laser, 600 nm and 775 nm depletion lasers, HC PL APO 100x/1.40 oil STED White objective, and gated HyD detectors. Images were further deconvolved using Huygens de-convolution software.

### Live cell imaging

Ibiditreat (Ibidi) 8-well chambers were used to seed Red F-actin-A549 cells or parental A549 cells at a density of 3,000 per chamber and incubated at 37°C overnight. Cells were infected with RSV-GFP, HPIV3-GFP or HMPV-GFP at an MOI of 0.1 unless mentioned otherwise for 1 hr at 37°C. Monolayers were washed 2x with only F-12 medium and incubated at 37°C in F-12 medium with 2% FBS and 1% L-glutamine and 25 mM HEPES for 24 hr prior to live cell imaging. For ARP2 knockdown, reverse transfections with siRNA were done 48 hr prior to infection with RSV-GFP in F-12 medium with 2% FBS, 1% L-glutamine and 25mM HEPES. Time-lapse images were acquired on an inverted Leica SP5 confocal equipped with a Ludin environmental chamber set to 37°C with 5% CO2, a HCX Pl APO 63X/1.4 oil objective, PMT detectors, Argon 488nm and DPSS 561nm lasers, and a motorized stage to enable the mark-and-find module in LAS AF software. Imaging started at 24 hpi and images were acquired approximately every 6 minutes, unless otherwise mentioned. Three randomly selected locations were imaged per sample per experiment. For imaging of the Red F-actin-A549 confluent monolayer, 30,000 cells were used and ARP2 knockdown was done with siARP2 for 48 hr before RSV-GFP infection (MOI = 0.1). To visualize nuclei in live cells, just before the start of the imaging, 50 nM Sir-DNA (Cytoskeleton Inc.) was added to the medium according to the manufacturer’s instructions. Imaging started at 24 hpi, and images were collected every 5 minutes for 24 hr on a Leica SP5 inverted confocal microscope equipped with a 63X/1.4NA objective, hybrid HyD detectors, an environmental chamber with CO_2_, and argon 488nm, DPSS 561nm, and HeNe 633nm lasers. Three randomly selected locations were imaged per sample.

### Scratch-wound assay

Reverse transfection with siARP2/ siControl was done for 48 hr on 30,000 Red F-actin-A549 cells in Ibiditreat 8-well chambers. Cells were mock-infected or infected with RSV-GFP at an MOI of 1 for 1 hr at 37°C. Monolayers were washed 2x with F-12 medium and incubated at 37°C in F-12 medium with 2% FBS and 1% L-glutamine and 25 mM HEPES for 24 hr. Cell monolayers were scratched with a 20 μl pipette tip (Molecular BioProducts), followed by imaging every 5 min for 12 hr on a Leica DMI6000 inverted wide-field microscope equipped with a 10x/0.4NA objective, a pco.edge sCMOS camera, adaptive focus control, and an environmental chamber with CO_2_. Three randomly selected locations were imaged per sample. Cell migration was measured by quantifying the intensity of Red F-actin in the scratch, normalized to the intensity of Red F-actin in the field at each time point using the image analysis software Imaris (Bitplane).

## Supporting Information

S1 Fig ARP2 knockdown reduced RSV production in Calu-3 cells. Calu-3 cells were transfected with siARP2, siControl or no siRNA for 48, 72, 96, or 120 hr. 48 hr after transfection cells were mock-infected or infected with RSV-WT (MOI = 1). **(A) ARP2 knockdown did not reduce cell viability.** Cell viability was compared using alamarBlue and expressed relative to the siControl. Data from two independent experiments, each done in triplicate were combined for analysis. Error bars: SD. **(B) ARP2 knockdown was stable.** ARP2 was detected similarly described in Fig 1A. **(C) ARP2 knockdown reduced RSV protein production.** RSV F was detected similarly as described in Fig 3A. **(D & E) ARP2 knockdown reduced production of infectious RSV.** Virus titers were measured in clarified tissue culture medium harvested from infected cell culture without disturbing the cell monolayer, **(D)** and virus titers were measured in clarified tissue culture medium from infected cell cultures in which the cells had been scraped into the medium and vortexed to release cell-associated virus (cell-associated virus plus released virus) **(E)**. D and E show combined data from two independent experiments, each performed in triplicate. Error bar: SD.
(TIF)

S2 Fig ARP2 knockdown has little effect on the release of HPIV3, and little effect on syncytium formation of RSV-infected cells. Replicate cultures of A549 cells were transfected with siARP2 or siControl for 48 hr, followed by infection with either RSV-GFP or HPIV3-GFP (MOI = 1). **(A) Effects of ARP2 knockdown on the titer of released HPIV3.** At 24, 48, and 72 hpi, the cell culture medium was harvested without disturbing the cells and clarified, and virus titers were determined by plaque assay with GFP staining (Materials and Methods). **(B) ARP2 knockdown has no effect on syncytium formation of RSV-infected cells.** The RSV-GFP-infected cell monolayers from the experiment in part A were fixed and permeabilized at the indicated time points, and F-actin was stained with rhodamine phalloidin and nuclei were stained with DAPI. The coverslips were imaged by confocal microscopy, and tiling was performed for an area of at least 5000 cells per coverslip (Materials and Methods). Within this area, the nuclei within GFP-positive cells (containing ≤2 nuclei) and GFP-positive syncytia (containing ≥3 nuclei) were counted, and the number of nuclei present in GFP-positive syncytia was divided by the total number of nuclei in GFP-positive cells and GFP-positive syncytium, and multiplied by 100:[(# nuclei in GFP-positive syncytia) / (# nuclei in GFP-positive cells and GFP-positive syncytia)] X 100. This was quantified in siARP2- and siControl-treated cells with RSV-GFP. The data in A and B were combined from two independent experiments, each performed in duplicate. Error bar: SD.
(TIF)

S3 Fig Evaluation of the surface of RSV-infected cells under SEM. A549 cells were transfected with siARP2 (panels 3, 4 and enlargements 4a) or siControl (panels 1, 2 and enlargements 2a). 48 hr post-transfection, cells were mock-infected (panels 1 and 3) or infected with RSV-GFP (MOI = 1, panels 2, 4, and magnified). At 24 hpi, cells were fixed with glutaraldehyde. Examples of filopodia on the presumptive RSV-GFP infected cells (compared with mock-infected cells) are indicated with cyan arrows.
(TIF)

S4 Fig RSV-induced filopodia are beta-tubulin-deficient. From the experiment shown in Fig 7, the panels here separately show staining for rhodamine phalloidin (*red*) to detect F-actin as a marker for filopodia, beta-tubulin (*cyan*, here a pseudocolor), RSV F protein (*green*), and a merge with the nuclear DAPI stain (*blue*). Filopodia are indicated with arrows.
(TIF)

S5 Fig N-WASP knockdown reduced RSV production in A549 cells. A549 cells were transfected with siN-WASP, siControl or no siRNA for 48, 72, 96, or 120 hr. 48 hr after transfection cells were mock-infected or infected with RSV-WT (MOI = 1). **(A) N-WASP knockdown had a modest effect on cell viability.** Cell viability was compared using alamarBlue and expressed relative to the siControl. Data obtained from three replicates of each sample. Error bars: SD. **(B) N-WASP knockdown was stable.** N-WASP was detected using a primary rabbit mAb and an anti-rabbit IgG IRDye800 secondary Ab. Alpha-tubulin, as a loading control, was detected with a primary mouse mAb and an anti-mouse IgG IRDye680 secondary Ab. **(C) N-WASP knockdown reduced RSV protein production.** RSV F was detected similarly described in Fig 3A. **(D & E) N-WASP knockdown reduced production of infectious RSV.** Virus titers were measured similarly described in Fig 5. Data obtained from three replicates of each sample. Error bar: SD.
(TIF)

S6 Fig N-WASP knockdown reduced RSV-induced filopodia. A549 cells were transfected with siN-WASP or siControl for 48 hr followed by mock infection or infection with RSV-WT (MOI = 1) for 24 hr. Cells were then fixed, permeabilized, and immunostained similarly as described in Figs 7 and S4. **(A)** RSV infected filopodia are shown in arrow. The number and length of filopodia were evaluated by automated scanning using confocal microscopy **(B).** In brief, Z-stacking for Alexafluor_488_ for RSV F protein, DAPI for nuclei, rhodamine phalloidin for F-actin was performed for 50 to 100 different random fields of interest in each coverslip. The length and number of filopodia was measured on 100 cells per treatment from the surface to the tip of the filopodium.
(TIF)

S1 Movie RSV-GFP infection in siControl-treated A549 cells. A549 cells were transfected with siControl for 48 hr followed by infection with RSV-GFP (MOI = 0.1). Cells were imaged every 6 min from 24 to 48 hpi. Time in hr: min: sec.
(MOV)

S2 Movie RSV-GFP infection in siARP2-treated A549 cells. A549 cells were transfected with siARP2 for 48 hr followed by infection with RSV-GFP (MOI = 0.1). Cells were imaged every 6 min from 24 to 48 hpi. Time in hr: min: sec.
(MOV)

S3 Movie Mock-infection in siControl-treated Red F-actin-A549 cells. Red F-actin-A549 cells (i.e. stably expressing a RFP engineered to bind to F-actin) were transfected with siControl for 48 hr followed by mock-infection. Cells were imaged every 6 min from 24 to 48 hpi. Time in hr: min: sec.
(MOV)

S4 Movie Mock-infection in siARP2-treated Red F-actin-A549 cells. Red F-actin-A549 cells were transfected with siARP2 for 48 hr followed by mock-infection. Cells were imaged every 6 min from 24 to 48 hpi. Time in hr: min: sec.
(MOV)

S5 Movie RSV-GFP infection in siControl-treated Red F-actin-A549 cells. Red F-actin-A549 cells were transfected with siControl for 48 hr followed by infection with RSV-GFP (MOI = 0.1). Cells were imaged every 6 min from 24 to 48 hpi. Time in hr: min: sec.
(MOV)

S6 Movie RSV-GFP infection in siARP2-treated Red F-actin-A549 cells. Red F-actin-A549 cells were transfected with siARP2 for 48 hr followed by infection with RSV-GFP (MOI = 0.1). Cells were imaged every 6 min from 24 to 48 hpi. Time in hr: min: sec.
(MOV)

S7 Movie Cell-to-cell spread of RSV-GFP in Red F-actin-A549 cells. Red F-actin-A549 cells were infected with RSV-GFP (MOI = 0.01). Cells were imaged every 6 min from 24 to 48 hpi. A magnified view of filopodia-driven RSV-GFP cell-to-cell spread. Time in hr: min: sec.
(MOV)

S8 Movie Mock-infection in siControl-treated Red F-actin-A549 confluent monolayer. Red F-actin-A549 cells were transfected with siControl for 48 hr followed by mock-infection. Cells were imaged every 5 min from 24 to 48 hpi. Time in hr: min: sec.
(MOV)

S9 Movie Mock-infection in siARP2-treated Red F-actin-A549 confluent monolayer. Red F-actin-A549 cells were transfected with siARP2 for 48 hr followed by mock-infection. Cells were imaged every 5 min from 24 to 48 hpi. Time in hr: min: sec.
(MOV)

S10 Movie RSV-GFP infection in siControl-treated Red F-actin-A549 confluent monolayer. Red F-actin-A549 cells were transfected with siControl for 48 hr followed by infection with RSV-GFP (MOI = 0.1). Cells were imaged every 5 min from 24 to 48 hpi. Time in hr: min: sec.
(MOV)

S11 Movie RSV-GFP infection in siARP2-treated Red F-actin-A549 confluent monolayer. Red F-actin-A549 cells were transfected with siARP2 for 48 hr followed by infection with RSV-GFP (MOI = 0.1). Cells were imaged every 5 min from 24 to 48 hpi. Time in hr: min: sec.
(MOV)

S12 Movie Infection of Red F-actin-A549 cells with RSV-GFP. Red F-actin-A549 cells were infected with the RSV-GFP (MOI = 0.1). Cells were imaged every 6 min from 24 to 48 hpi. Time in hr: min: sec.
(MOV)

S13 Movie Infection of Red F-actin-A549 cells with HPIV3-GFP. Red F-actin-A549 cells were infected with the HPIV3-GFP (MOI = 0.1). Cells were imaged every 6 min from 24 to 48 hpi. Time in hr: min: sec.
(MOV)

S14 Movie Infection of Red F-actin-A549 cells with HMPV-GFP. Red F-actin-A549 cells were infected with the HMPV-GFP (MOI = 0.1). Cells were imaged every 6 min from 24 to 48 hpi. Time in hr: min: sec.
(MOV)
