# Supplementary figures and images for: Actin-Related Protein 2 (ARP2) and Virus-Induced Filopodia Facilitate Human Respiratory Syncytial Virus Spread
Source: PLoS Pathog. 2016 Dec 7;12(12):e1006062. doi: 10.1371/journal.ppat.1006062 (PMC5142808; doi:10.1371/journal.ppat.1006062)

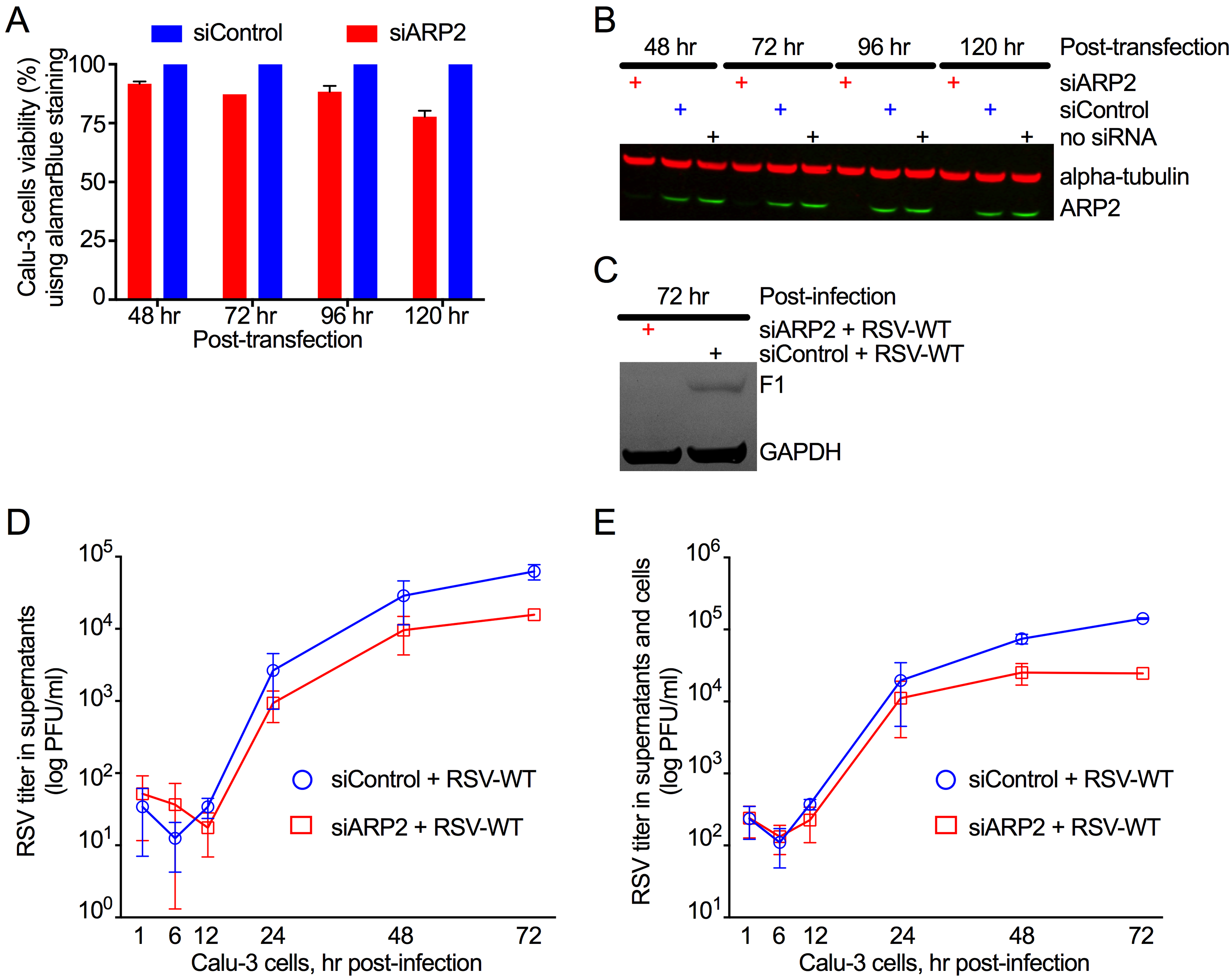

Supplement: S1 Fig — Calu-3 cells were transfected with siARP2, siControl or no siRNA for 48, 72, 96, or 120 hr. 48 hr after transfection cells were mock-infected or infected with RSV-WT (MOI = 1). (A) ARP2 knockdown did not reduce cell viability. Cell viability was compared using alamarBlue and expressed relative to the siControl. Data from two independent experiments, each done in triplicate were combined for analysis. Error bars: SD. (B) ARP2 knockdown was stable. ARP2 was detected similarly described in Fig 1A. (C) ARP2 knockdown reduced RSV protein production. RSV F was detected similarly as described in Fig 3A. (D & E) ARP2 knockdown reduced production of infectious RSV. Virus titers were measured in clarified tissue culture medium harvested from infected cell culture without disturbing the cell monolayer, (D) and virus titers were measured in clarified tissue culture medium from infected cell cultures in which the cells had been scraped into the medium and vortexed to release cell-associated virus (cell-associated virus plus released virus) (E). D and E show combined data from two independent experiments, each performed in triplicate. Error bar: SD. (TIF) [file ppat.1006062.s001.tif]

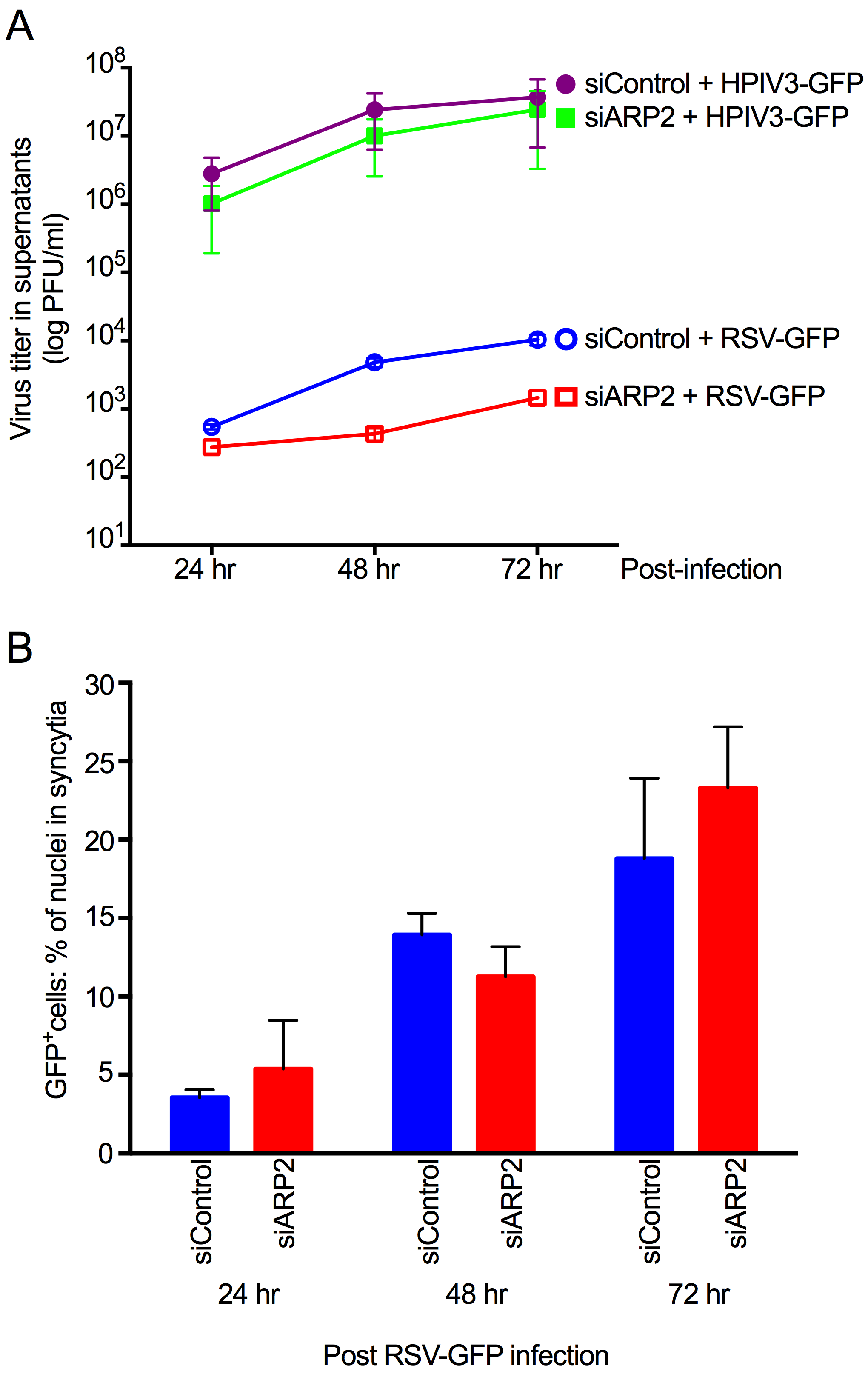

Supplement: S2 Fig — Replicate cultures of A549 cells were transfected with siARP2 or siControl for 48 hr, followed by infection with either RSV-GFP or HPIV3-GFP (MOI = 1). (A) Effects of ARP2 knockdown on the titer of released HPIV3. At 24, 48, and 72 hpi, the cell culture medium was harvested without disturbing the cells and clarified, and virus titers were determined by plaque assay with GFP staining (Materials and Methods). (B) ARP2 knockdown has no effect on syncytium formation of RSV-infected cells. The RSV-GFP-infected cell monolayers from the experiment in part A were fixed and permeabilized at the indicated time points, and F-actin was stained with rhodamine phalloidin and nuclei were stained with DAPI. The coverslips were imaged by confocal microscopy, and tiling was performed for an area of at least 5000 cells per coverslip (Materials and Methods). Within this area, the nuclei within GFP-positive cells (containing ≤2 nuclei) and GFP-positive syncytia (containing ≥3 nuclei) were counted, and the number of nuclei present in GFP-positive syncytia was divided by the total number of nuclei in GFP-positive cells and GFP-positive syncytium, and multiplied by 100:[(# nuclei in GFP-positive syncytia) / (# nuclei in GFP-positive cells and GFP-positive syncytia)] X 100. This was quantified in siARP2- and siControl-treated cells with RSV-GFP. The data in A and B were combined from two independent experiments, each performed in duplicate. Error bar: SD. (TIF) [file ppat.1006062.s002.tif]

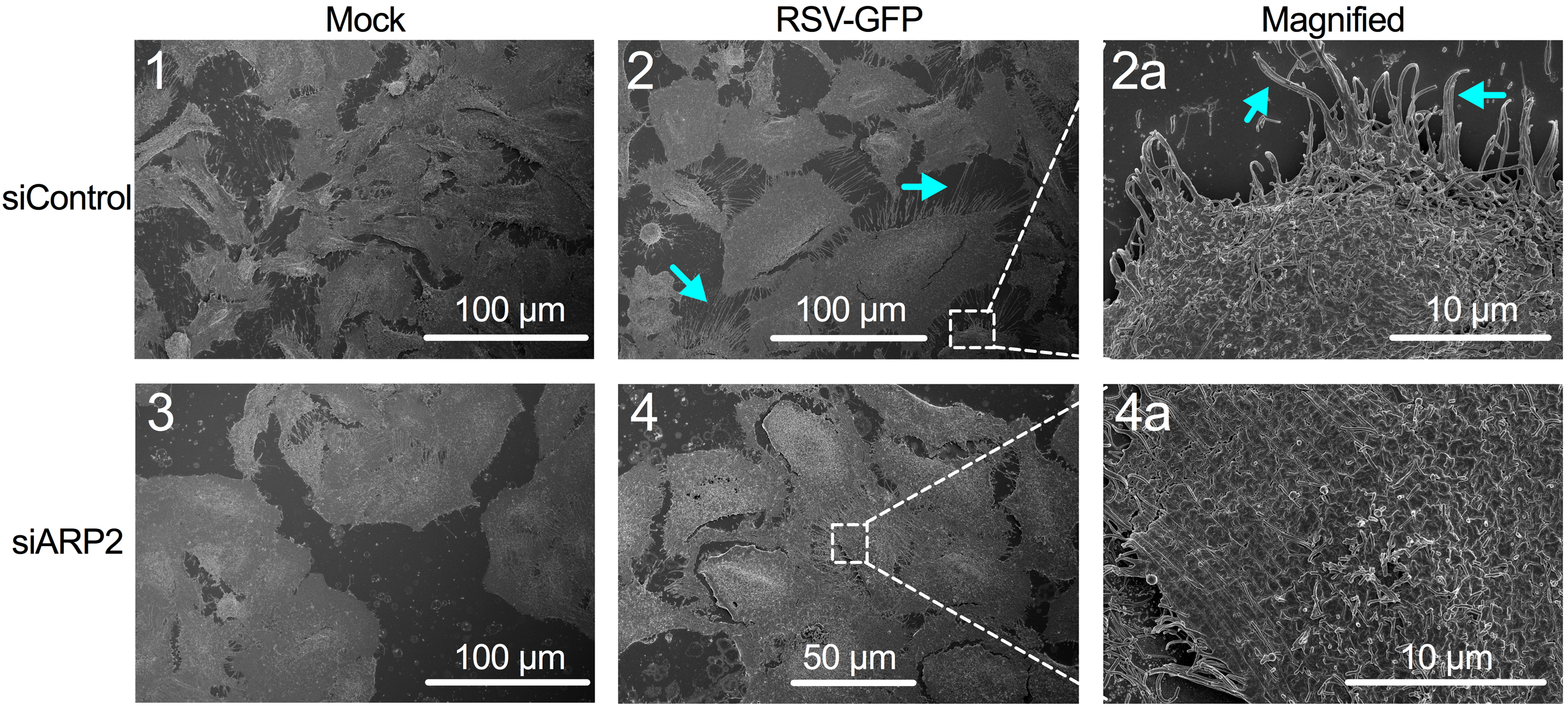

Supplement: S3 Fig — A549 cells were transfected with siARP2 (panels 3, 4 and enlargements 4a) or siControl (panels 1, 2 and enlargements 2a). 48 hr post-transfection, cells were mock-infected (panels 1 and 3) or infected with RSV-GFP (MOI = 1, panels 2, 4, and magnified). At 24 hpi, cells were fixed with glutaraldehyde. Examples of filopodia on the presumptive RSV-GFP infected cells (compared with mock-infected cells) are indicated with cyan arrows. (TIF) [file ppat.1006062.s003.tif]

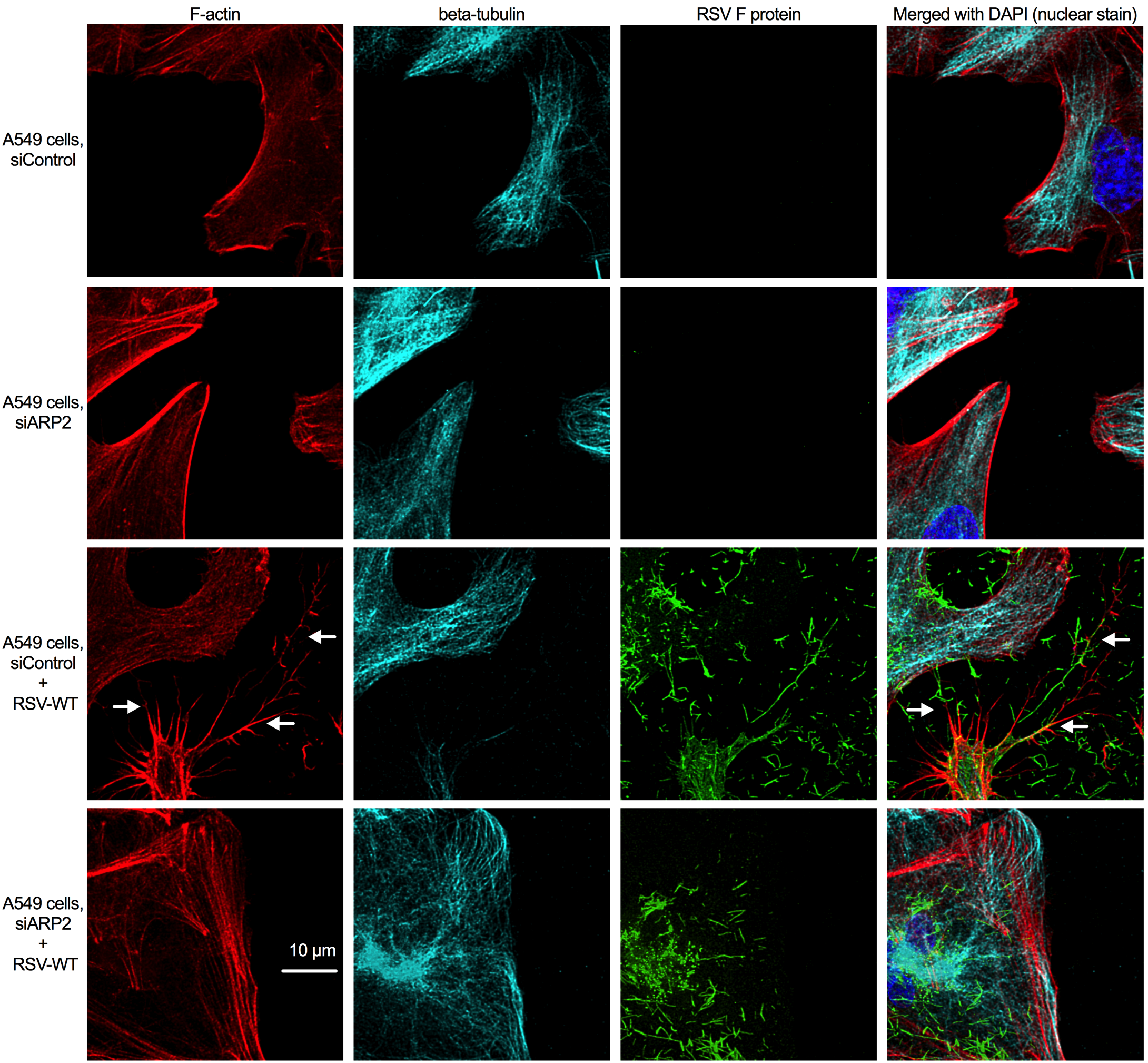

Supplement: S4 Fig — From the experiment shown in Fig 7, the panels here separately show staining for rhodamine phalloidin (red) to detect F-actin as a marker for filopodia, beta-tubulin (cyan, here a pseudocolor), RSV F protein (green), and a merge with the nuclear DAPI stain (blue). Filopodia are indicated with arrows. (TIF) [file ppat.1006062.s004.tif]

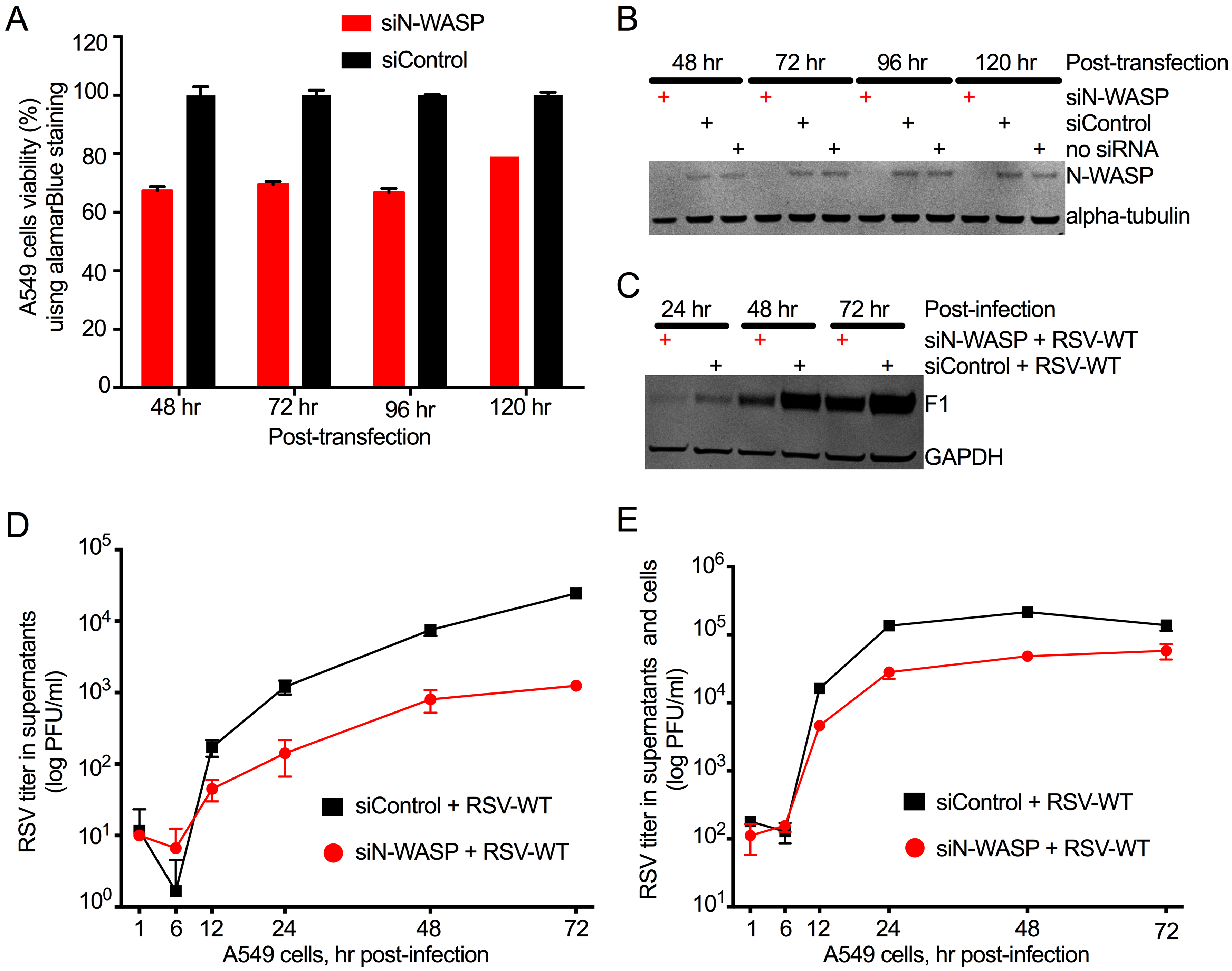

Supplement: S5 Fig — A549 cells were transfected with siN-WASP, siControl or no siRNA for 48, 72, 96, or 120 hr. 48 hr after transfection cells were mock-infected or infected with RSV-WT (MOI = 1). (A) N-WASP knockdown had a modest effect on cell viability. Cell viability was compared using alamarBlue and expressed relative to the siControl. Data obtained from three replicates of each sample. Error bars: SD. (B) N-WASP knockdown was stable. N-WASP was detected using a primary rabbit mAb and an anti-rabbit IgG IRDye800 secondary Ab. Alpha-tubulin, as a loading control, was detected with a primary mouse mAb and an anti-mouse IgG IRDye680 secondary Ab. (C) N-WASP knockdown reduced RSV protein production. RSV F was detected similarly described in Fig 3A. (D & E) N-WASP knockdown reduced production of infectious RSV. Virus titers were measured similarly described in Fig 5. Data obtained from three replicates of each sample. Error bar: SD. (TIF) [file ppat.1006062.s005.tif]

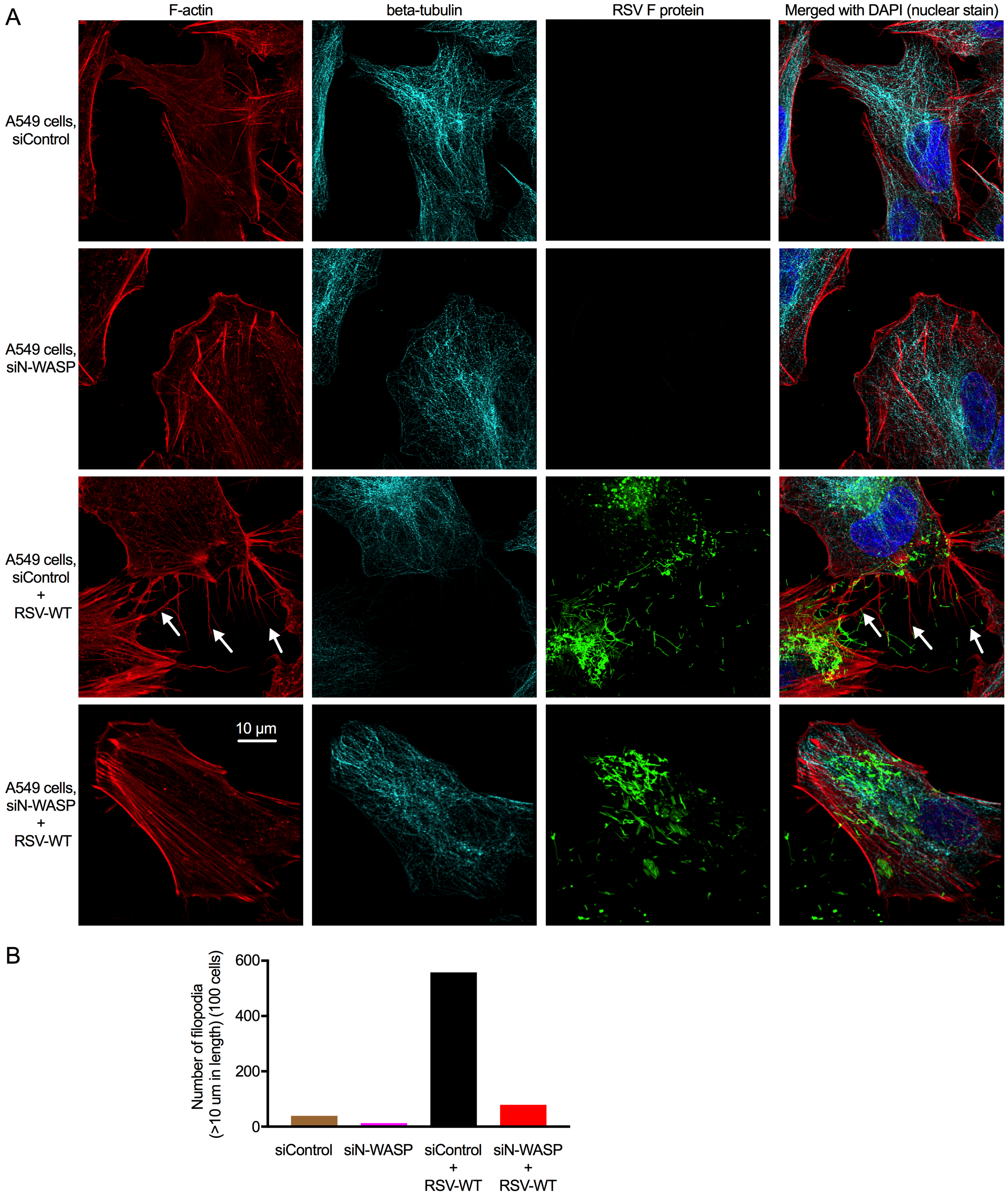

Supplement: S6 Fig — A549 cells were transfected with siN-WASP or siControl for 48 hr followed by mock infection or infection with RSV-WT (MOI = 1) for 24 hr. Cells were then fixed, permeabilized, and immunostained similarly as described in Figs 7 and S4. (A) RSV infected filopodia are shown in arrow. The number and length of filopodia were evaluated by automated scanning using confocal microscopy (B). In brief, Z-stacking for Alexafluor488 for RSV F protein, DAPI for nuclei, rhodamine phalloidin for F-actin was performed for 50 to 100 different random fields of interest in each coverslip. The length and number of filopodia was measured on 100 cells per treatment from the surface to the tip of the filopodium. (TIF) [file ppat.1006062.s006.tif]
